# The CK2 Kinase Stabilizes CLOCK and Represses Its Activity in the *Drosophila* Circadian Oscillator

**DOI:** 10.1371/journal.pbio.1001645

**Published:** 2013-08-27

**Authors:** Áron Szabó, Christian Papin, Daniela Zorn, Prishila Ponien, Frank Weber, Thomas Raabe, François Rouyer

**Affiliations:** 1 Institut de Neurobiologie Alfred Fessard, Centre National de la Recherche Scientifique Unité Propre de Recherche 3294, Gif-sur-Yvette, France; 2 Département de Biologie, Université Paris Sud, Orsay, France; 3 Heidelberg University, Biochemistry Center (BZH), Im Neuenheimer Feld 328, Heidelberg, Germany; 4 Institut de Chimie des Substances Naturelles, CNRS UPR2301, Gif-sur-Yvette, France; 5 IMAGIF, Centre de Recherche de Gif, Gif-sur-Yvette, France; 6 University of Wuerzburg, Institute of Medical Radiation and Cell Research, Wuerzburg, Germany; University of Geneva, Switzerland

## Abstract

Phosphorylation is a pivotal regulatory mechanism for protein stability and activity in circadian clocks regardless of their evolutionary origin. It determines the speed and strength of molecular oscillations by acting on transcriptional activators and their repressors, which form negative feedback loops. In *Drosophila*, the CK2 kinase phosphorylates and destabilizes the PERIOD (PER) and TIMELESS (TIM) proteins, which inhibit CLOCK (CLK) transcriptional activity. Here we show that CK2 also targets the CLK activator directly. Downregulating the activity of the catalytic α subunit of CK2 induces CLK degradation, even in the absence of PER and TIM. Unexpectedly, the regulatory β subunit of the CK2 holoenzyme is not required for the regulation of CLK stability. In addition, downregulation of CK2α activity decreases CLK phosphorylation and increases *per* and *tim* transcription. These results indicate that CK2 inhibits CLK degradation while reducing its activity. Since the CK1 kinase promotes CLK degradation, we suggest that CLK stability and transcriptional activity result from counteracting effects of CK1 and CK2.

## Abstract 2

The CK2 kinase plays diverse roles in the *Drosophila* circadian clock by controlling stability of the CLOCK transcription factor itself, besides its known role in degrading CLOCK repressors.

## Author Summary

The CK2 kinase is an ancient enzyme known to be at the heart of self-sustaining circadian clocks in animals, plants, and fungi. Circadian clocks are responsible for daily circadian rhythms in molecular, physiological, and behavioral processes. Their mechanism relies on transcriptional activators and repressors that constitute a feedback loop. The CLOCK (CLK) activator is required to initiate the transcription of the *period* (*per*) and *timeless* (*tim*) genes in the late day. The PER and TIM repressors accumulate in a delayed manner and translocate to the nucleus to repress their own gene. Degradation of these repressors allows the activator to start a new cycle. In the fruit fly *Drosophila melanogaster*, CK2-mediated phosphorylation of the PER and TIM repressors targets them for degradation. Here we find that the CLK activator is also regulated by CK2. In contrast to PER and TIM, CLK is stabilized by CK2α phosphorylation; we also show that PER and TIM are dispensable for this stabilization of CLK. The identification of CK2–CLK protein complexes and the *in vitro* phosphorylation of CLK by CK2 hint at a direct action of the kinase on the activator. Although transcription factor stabilization is generally expected to be associated with increased activity, genetic analysis indicates that CK2 represses CLK. Hence CK2 phosphorylation seems to be a key signal for the transcriptional activator complex to adopt a proper activation state at a given point of the circadian cycle.

## Introduction

Circadian oscillations of gene expression, physiology, and behavior are found in a wide range of organisms. They are governed by temporally regulated feedback loops in which transcription factors activate the expression of their own inhibitors. In the *Drosophila* circadian oscillator, the CLOCK (CLK) and CYCLE (CYC) bHLH-PAS domain transcription factors activate expression of the *period* (*per*) and *timeless* (*tim*) genes at the end of the day. The delayed accumulation of PER and TIM and their transfer to the nucleus leads to transcriptional repression of CLK/CYC during the late night. The repression phase is also shaped by other repressors/activators such as CLOCKWORK ORANGE (CWO) and KAYAK-α [1]. Subsequent degradation of PER and TIM repressors in the morning allows transcription to resume towards the evening [2],[3]. Controlled phosphorylation, ubiquitylation, and proteasome-dependent degradation of PER and TIM set the timing of their delayed accumulation and clearance. The PER protein is phosphorylated by the DOUBLETIME (DBT, CK1δ/ε), CK2, and NEMO kinases and polyubiquitylated by the SCF^Slimb^ ubiquitin ligase complex [4]–[12]. TIM associates with PER, preventing its degradation, but TIM itself is subjected to phosphorylation and subsequent breakdown. TIM phosphorylation involves the CK2 and SHAGGY (SGG, GSK-3) kinases and TIM degradation also depends on SCF^Slimb^ and a CULLIN-3-based ubiquitin ligase complex [7],[8],[13]–[15]. Phosphatase activity counterbalances the effects of the aforementioned and probably also of other kinases: PP2A regulates PER abundance, while PP1 targets both PER and TIM [16],[17].

CLK phosphorylation cycles with a peak in the morning and a minimum in the early night [18]–[21]. Similarly, CLK immunoreactivity in head extracts or brain tissue seems to oscillate in phase with its phosphorylation [21]–[23], although harsh extraction liberates chromatin-bound CLK, which results in relatively constant CLK levels [20],[24],[25]. Whether oscillations of CLK immunoreactivity in neurons reflect rhythmic changes of total CLK protein amount is still unclear [23],[26]. Due to the cyclic regulation of CLK as opposed to constitutive expression of CYC, the CLK protein appears to represent the key rhythmic component of the circadian activator in *Drosophila* [27]. CLK DNA-binding and transcriptional activity show a robust oscillation with an evening peak that is associated with the rapid increase of *per* and *tim* mRNA levels [20],[22]. The release of CLK from DNA goes hand in hand with its hyperphosphorylation, which depends on both PER and DBT [19],[20],[22]. Since kinase activity of DBT does not seem to be required for hyperphosphorylation, it was proposed that DBT acts as an interface for the recruitment of other kinases into a complex with CLK [28]. The PER kinase NEMO destabilizes CLK *in vivo* and might thus be a CLK kinase [29]. CLK transcriptional activity in cultured cells is affected by calcium/calmodulin-dependent kinase II and mitogen-activated protein kinase [30]. Ubiquitylation is also involved in the regulation of CLK and BMAL1, the CYC ortholog in mammals [31],[32]. In Drosophila, USP8 was recently reported to decrease CLK activity by deubiquitylation [25].

The CK2 kinase has a key function in the clockwork of various organisms [33]. In *Neurospora*, CK1 and CK2 phosphorylate both the White Collar Complex (WCC) transcriptional activator as well as its inhibitor FREQUENCY (FRQ) to control their activity, subcellular localization, and stability [34]–[36]. In mammals, CK2 and CK1 destabilize PER2, although phosphorylation at specific CK2 target sites stabilizes the protein [37],[38]. The CK2 holoenzyme is formed by a tetrameric complex consisting of two catalytic (α) and two interacting regulatory (β) subunits [39]. The β subunits stabilize the α subunits that possess constitutive kinase activity. Phosphorylation of most substrates is enhanced by CK2β, while some substrates are more efficiently phosphorylated by free CK2α in the absence of CK2β [40]. In *Drosophila*, CK2α and CK2β affect PER and TIM abundance and subcellular localization, which correlates with a direct phosphorylation of both proteins by the CK2 holoenzyme *in vitro* [8],[9],[41]–[43]. The dominant-negative CK2α^Tik^ mutation strongly increases TIM stability even in the absence of PER, supporting TIM as the main target of CK2 [15]. The CK2α^Tik^ protein overexpression induces hyperphosphorylation of TIM that could be explained by enhanced phosphorylation or reduced dephosphorylation of TIM by other kinases and phosphatases [15].

Since the identity of the kinases involved in the control of CLK phosphorylation remains unclear, we asked whether CK2 plays a role in the phosphorylation and regulation of CLK. Our results indicate that inhibition of CK2α activity strongly increases CLK degradation, whereas CK2β does not affect CLK stability. The CK2 holoenzyme is recruited onto PER, TIM, and CLK mainly during late night, inducing CLK hyperphosphorylation *in vivo* and CK2 phosphorylates CLK *in vitro.* Specific CLK activity is increased in dominant-negative CK2α^Tik^-expressing flies indicating repression of CLK by CK2α. Our findings define, to our knowledge, the first *bona fide* kinase of *Drosophila* CLK that plays a role in its degradation and hyperphosphorylation. The unstable but strongly active CLK acquired by CK2α inhibition joins the club of other circadian transcription factors with similar properties such as the WCC complex in *Neurospora*.

## Results

### CK2α Activity Promotes CLK Protein Phosphorylation and Stability

**Figure 1:**
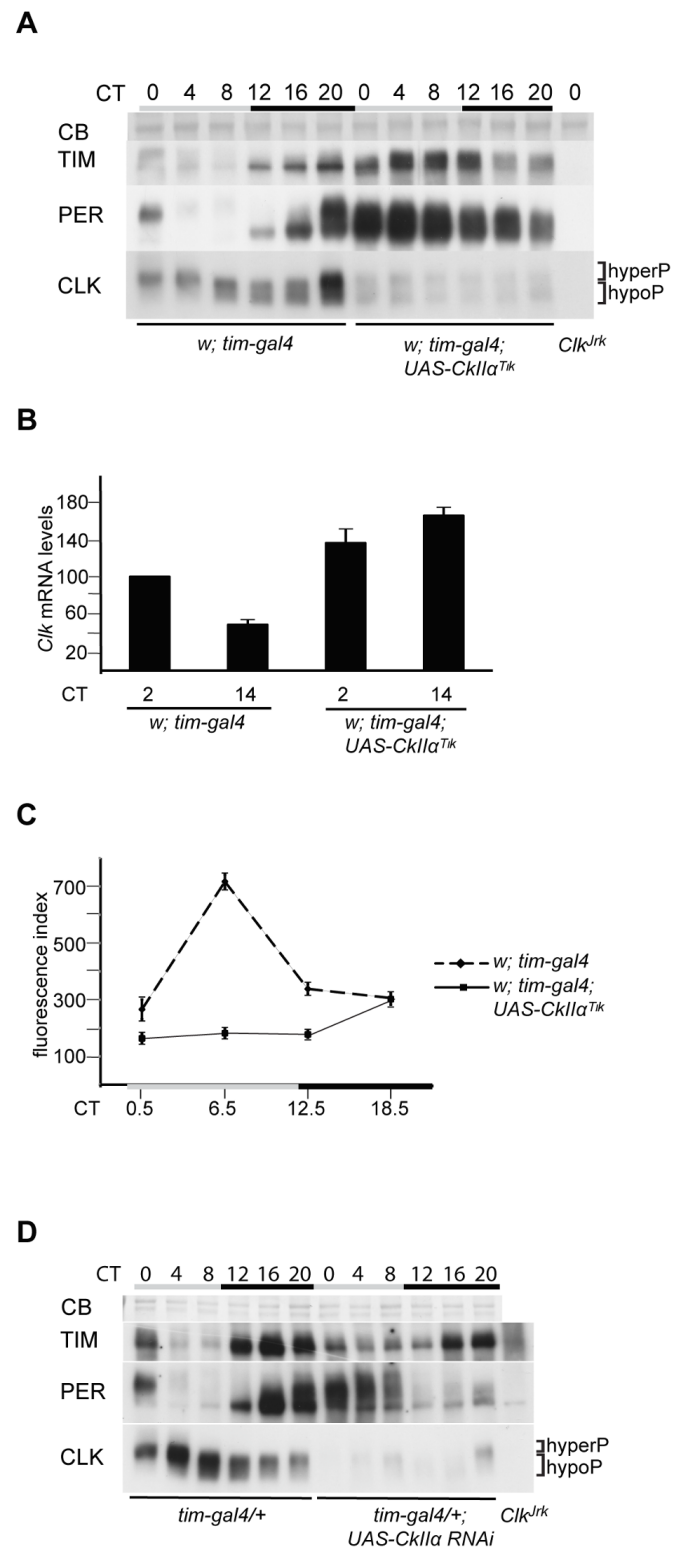
CK2α inhibition triggers CLK degradation. (A, D) Western blot of sonicated head extracts from flies collected at DD1. Time (h) is indicated as CT. Gray and black bars represent subjective day and subjective night, respectively. A Coomassie Blue (CB) stained band in the size range of CLK is used as a loading control for blots run on 4% gels. Brackets indicate hypo- and hyperphosphorylated forms of CLK. At least two independent experiments were performed for each blot. (A) Two copies of *tim-gal4* and two copies of *UAS-CkIIα^Tik^* transgene were used for the experimental genotype. (B) Quantitative RT-PCR measurements of *Clk* mRNA levels in heads of flies collected at DD1. Results were averaged from at least four independent experiments. Error bars indicate the s.e.m. Averaged values were normalized to the CT2 control averaged value set to 100. (C) Quantification of CLK immunofluorescence in the PDF-expressing s-LNvs. Fluorescence index is given in arbitrary units. Error bars indicate s.e.m. (D) Flies were entrained and collected at 29°C. One copy of *tim-gal4* and two copies of the *CkIIα* RNAi construct were used for the experimental genotype.

A putative role of CK2 in CLK regulation was first addressed by analyzing head extracts of flies expressing a dominant-negative version of the CK2α catalytic subunit. As previously reported [15],[42], *w;tim-gal4;UAS-CkIIα^Tik^* flies (hereafter *tim>Tik* flies) were behaviorally arrhythmic (Table 1) and displayed weak and strongly delayed PER and TIM oscillations, with high levels of mildly phosphorylated PER and highly phosphorylated TIM (Figure 1A). As CLK efficiently binds to DNA in the evening, the estimation of CLK levels through the circadian cycle is affected by extraction conditions. In sonicated head extracts, CLK protein has been shown to stay at constant levels, in contrast to a robust cycle of its phosphorylation [19],[20]. However, the existence of oscillations in CLK levels remains discussed [22]–[25]. In our hands, sonicated extracts of control flies showed weak cycling of CLK levels, although peak time was rather variable between experiments (Figures 1A and S1A). Nonsonicated extracts always showed CLK levels cycling with a trough in the evening (Figure S1B). Importantly, both sonicated and nonsonicated extracts of *tim>Tik* flies showed very low CLK levels with reduced phosphorylation on the first day of constant darkness (DD) (Figures 1A and S1A and S1B). In order to better estimate CLK levels in *tim>Tik* flies, sonicated extracts were treated with λ protein phosphatase (Figure S1C). Again, a strong decrease of unphosphorylated CLK abundance was observed in *tim>Tik* animals. Moreover, *Clk* mRNA levels were about 1.5-fold higher in *tim>Tik* flies than in controls (Figure 1B), indicating that low CLK protein levels are not a consequence of reduced *Clk* expression. Consequently, the protein/mRNA ratio for CLK decreased to approximately 10% in *tim>Tik* (Figure S1D). Immunolabeling of whole-mount brains of *tim>Tik* flies also supported a strong reduction of CLK levels in the small ventral lateral neurons (s-LNvs) (Figure 1C), with no change in its nuclear-only localization (not shown).

**Table 1:** Locomotor activity rhythms in constant darkness of flies.

Genotype	Number of flies	Rhythmic flies (%)	Period length (h)	Power
*w; tim-GAL4/+*	12	100	23.6±0.1	198±13
*yw;; UAS-CkIIα^Tik^*	16	81	24.0±0.1	72±13
*w; tim-GAL4/+;UAS-CkIIα^Tik^/+*	21	100	30.9±0.1	178±8
*w; tim-GAL4;UAS-CkIIα^Tik^*	16	18*	27.8±2.9	31±3
*w; UAS-CkIIα/+*	9	88	24.1±0.1	144±22
*w; tim-GAL4/UAS-CkIIα*	16	100	25.6±0.1	211±9
*w;; UAS-CkIIα RNAi 17520 R-2*	14	92	24.8±0.1	133±13
*w; tim-GAL4/CyO; UAS-CkIIα RNAi 17520 R-2*	14	93	32.1±0.3	143±10
*w; UAS-FLAG-CkIIα/+*	16	100	23.6±0.1	185±17
*w; tim-GAL4/UAS-FLAG-CkIIα*	8	100	24.6±0.1	142±19
*w; UAS-CkIIβ RNAi #106845/+; UAS-CkIIβ RNAi #32377/+*	14	100	23.4±0.1	207±16
*w; tim-GAL4/UAS-CkIIβ RNAi #106845; UAS-CkIIβ RNAi #32377/+*	26	42*	31.2±1.6	34±5
*w;; gal1118/+*	15	100	24.4±0.1	187±8
*w;; gal1118/UAS-CkIIβ RNAi #32377*	29	93	33.1±0.3	89±8
*w; UAS:CkIIβ-VIIb(4)/+*	15	86	24.5±0.1	145±14
*w; UAS:CkIIβ-VIIb(5)/+*	14	92	24.2±0.1	129±15
*w; UAS:CkIIβ-VIIc(6)/+*	16	93	23.8±0.2	141±14
*w; UAS:CkIIβ-VIIa(5)/+;gal1118/UAS-CkIIβ RNAi #32377*	17	100	24.8±0.1	220±10
*w; UAS:CkIIβ-VIIb(4)/+;gal1118/UAS-CkIIβ RNAi #32377*	24	100	24.5±0.1	215±6
*w; UAS:CkIIβ-VIIb(5)/+;gal1118/UAS-CkIIβ RNAi #32377*	16	100	25.0±0.1	217±7
*w; UAS:CkIIβ-VIIc(2)/+;gal1118/UAS-CkIIβ RNAi #32377*	15	100	25.4±0.1	226±10
*w; UAS:CkIIβ-VIIc(6)/+;gal1118/UAS-CkIIβ RNAi #32377*	16	100	25.3±0.1	220±6
*w; UAS-gfp/+;gal1118/+*	16	100	23.6±0.1	180±15
*w; UAS-gfp/+;gal1118/UAS-CkIIβ RNAi #32377*	30	90	32.5±0.1	85±8
*w; UAS-FLAG-CkIIβ/+*	13	92	24.0±0.3	138±17
*w; tim-GAL4/UAS-FLAG-CkIIβ*	15	100	24.7±0.1	166±14
*w; UAS-FLAG-CkIIβ/+;gal1118/UAS-CkIIβ RNAi #32377*	14	93	24.6±0.1	219±22

The mean values of period (in hours) and associated power (see Materials and Methods) are given ± s.e.m.

* Genotypes considered arrhythmic (see Materials and Methods).

To independently analyze the effect of decreasing CK2α activity on CLK, a UAS-*CkIIα* RNA interference (RNAi) construct was expressed under *tim-gal4* control. Adult flies were kept at 29°C to increase Gal4-dependent expression. CLK in sonicated head extracts of *w;tim-gal4/+;UAS-CkIIα-RNAi* (*tim>CkIIα-RNAi*) flies showed a similar phenotype to that of *tim>Tik* flies, with reduced phosphorylation and levels throughout the cycle (Figure 1D). Dampened and delayed PER and TIM oscillations were observed with increased protein levels during the day. In support of its specificity, the induction of RNAi reduced CK2α protein levels (Figure S1E). Although high mortality of *tim>CkIIα-RNAi* flies after long incubation at high temperature prevented the assessment of their locomotor activity rhythms at 29°C, they displayed long period rhythms at 25°C (Table 1). Similar long period rhythms are observed in heterozygous *w;tim-gal4/+;UAS-CkIIα^Tik^/+* flies (Table 1), as previously reported [42].

### CK2α Stabilizes CLK in the Absence of PER and TIM

**Figure 2:**
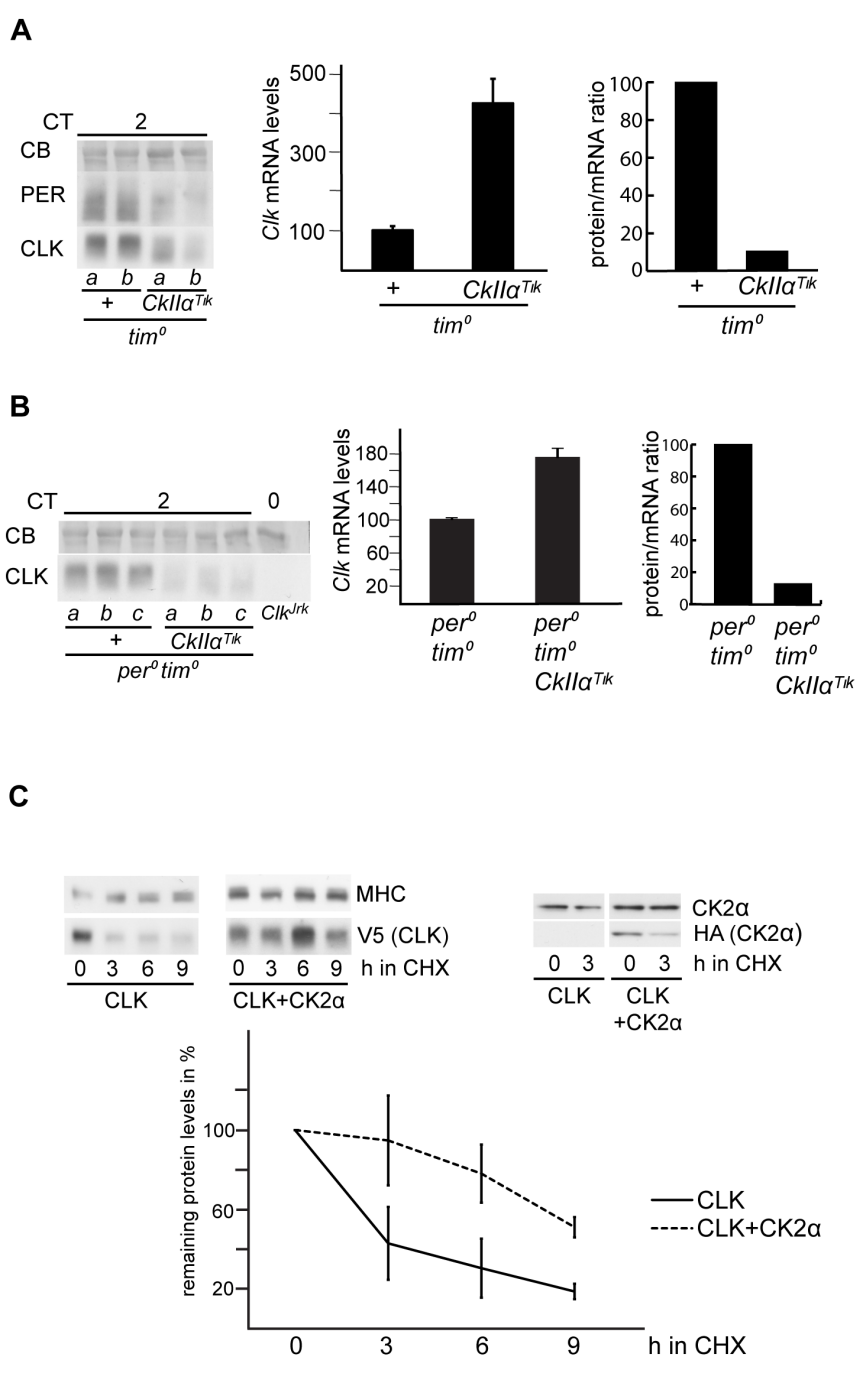
PER and TIM-independent degradation of CLK in CK2α^Tik^ overexpressing flies. (A, B) Western blot of sonicated head extracts from flies collected at DD1. A CB stained band in the size range of CLK is used as a loading control. At least two independent experiments were performed for each blot. (A) Comparison of CLK protein in *tim>Tik* and control flies in *tim^0^* background. (Left) Comparison between *tim>Tik* and controls in *tim^0^* background for PER and CLK at CT2. *w; tim^0^ tim-gal4* (*tim^0^* +) and *w; tim^0^ tim-gal4; UAS-CkIIα^Tik^* (*tim^0^ CkIIα^Tik^*) were used. *a* and *b* are different protein extracts from the same genotype at the same time point. We loaded 100 µg of extracts. (Middle) Quantitative RT-PCR measurements of *Clk* mRNA levels in heads of flies collected at DD1. Results are means of pooled values from two time points (CT2 and 14) with at least two independent samples for each time point. Error bars indicate s.e.m. Average values were normalized to the mean of the control (*w; tim^0^ tim-gal4*) set to 100. Previous analysis of separate values at CT2 and CT14 indicated that they were similar (Table S1) justifying their common treatment (see above). (Right) CLK protein/*Clk* mRNA ratio calculated from mean CT2–CT14 values of Western blot quantification and quantitative RT-PCR data. Ratios were normalized to the control (*w; tim^0^; tim-gal4*) set to 100. Abbreviations as in (A). (Right) CLK protein/*Clk* mRNA ratio calculated from mean CT2–CT14 values of Western blot quantification and quantitative RT-PCR data. Ratios were normalized to the control (*w; tim^0^; tim-gal4*) set to 100. (B) Comparison of CLK protein in *tim>Tik* and control flies in *per^0^ tim^0^* background. (Left) Genotypes: *per^0^ w*; *tim^0^* (*per^0^ tim^0^*) and *per^0^ w*; *tim^0^ tim-gal4; UAS-CkIIα^Tik^* (*per^0^ tim^0^ CkIIα^Tik^*) as well as *w*;;*Clk^Jrk^*. *a*, *b*, and *c* are different protein extracts from the same genotype at the same time point. (Middle) Quantitative RT-PCR measurements of *Clk* mRNA levels in head extracts of flies collected at CT2. Mean values +/− s.e.m. from at least three independent experiments are shown with the *per^0^ tim^0^* control set to 100. (Right) CLK protein/*Clk* mRNA ratio calculated with mean values of Western blot quantification and quantitative RT-PCR data at CT2. Ratios were normalized to the control (*per^0^ tim^0^*) set to 100. (C) Cycloheximide chase of CLK degradation in the presence of CK2α overexpression. *per* and *tim* dsRNA was applied to S2 cells prior to transfection. We transfected 1 µg pAc-*Clk*-V5/His6 with or without 3 µg of the FMO02931 CK2α expression vector. Transfections were split in four equal volumes for the degradation assay. “h in CHX” indicates the hours for which respective cells were incubated in cycloheximide to stop protein synthesis. Immunoreactivity against MHC (myosin heavy chain) was used as loading control. Anti-V5, anti-CK2α, and anti-HA were used to reveal CLK and CK2α, respectively. (Left) Western blots for CLK alone (CLK) and CLK cotransfected with CK2α (CLK+ CK2α). (Right) CK2α expression from the FMO02931 plasmid after 1 d of induction. (Bottom) Average degradation profiles from three independent experiments (as shown on the left). Error bars represent s.e.m.

TIM was described as the likely primary target of CK2α in the circadian clock, effects elicited on PER being only secondary [15]. We thus asked whether TIM was required for CK2α effects on CLK, by comparing the profile of CLK protein in sonicated head extracts of *tim^01^* and *tim^01^ tim>Tik* flies. In the absence of TIM, the CK2α^Tik^ protein induced a prominent reduction in CLK phosphorylation and a significant decrease of protein levels (Figure 2A). Furthermore, *Clk* mRNA levels were about four times higher in *tim^0^ tim>Tik* compared to *tim^0^*, supporting a strong degradation of the CLK protein in *tim^0^ tim>Tik* flies (Figure 2A). Since a PER/DBT complex controls CLK phosphorylation [20],[28], we asked whether PER was required for CLK modifications by CK2α, even in the absence of TIM. Effects of the CK2α^Tik^ protein were thus analyzed in *per^0^ tim^0^* double mutants, where CLK appeared minimally phosphorylated in a *CkIIα* ^+^ background (Figures 2B and S2A). CLK levels and phosphorylation were further diminished in the presence of the CK2α^Tik^ protein (Figures 2B and S2A). Since the CK2α^Tik^ protein overproduction increased *Clk* mRNA levels by about twofold in *per^0^ tim^0^* double mutants, the CLK protein/mRNA ratio was reduced just as in *tim^0^* mutants (Figure 2B). A similar decrease of CLK protein levels was observed in *per^0^ tim>Tik* flies (Figure S2B) despite increased *Clk* mRNA levels, suggesting that CLK protein was again strongly destabilized in the absence of PER. These observations reveal that CK2α stabilizes CLK in the absence of PER and/or TIM. Since CLK phosphorylation is further decreased by CK2α^Tik^ expression, CK2α is important for the PER/TIM-independent minimal phosphorylation program of CLK.

The up-regulation of *Clk* mRNA levels in *tim>Tik* flies suggested that CK2α could repress *Clk* transcription. To test whether *Clk* transcription was affected in the *tim>Tik* genotype, *Clk* pre-mRNA levels were estimated. They were not increased in *tim>Tik* flies compared to controls, although a reduced antiphasic cycling was observed at DD1 (Figure S2C). The antiphasic oscillation was reminiscent of PER and TIM oscillations persisting in these flies (see Figure 1A). The increase of mature *Clk* mRNA levels in *tim>Tik* flies thus seems not to be the consequence of higher *Clk* gene transcription and rather supports a posttranscriptional control of *Clk* mRNA by CK2α. In agreement with a posttranscriptional control, the VRI and PDP1 regulators of *Clk* transcription were not affected in *per^0^ tim>Tik* flies (Figure S2D). Finally, since the transcriptional regulation of the *cryptochrome* (*cry*) gene is similar to the one of the *Clk* gene [44], we tested *cry* mRNA levels in *per^0^ tim>Tik* flies. No increase of *cry* mRNA levels was observed in the presence of the CK2α^Tik^ protein (Figure S2E), supporting a specific control of *Clk* mRNA levels by CK2α.

The data from *tim>Tik* flies strongly suggested that CK2α controls CLK stability independently from PER and TIM. To obtain direct evidence for this, CLK degradation kinetics were analyzed in a cycloheximide (CHX) chase-based assay in Drosophila Schneider 2 (S2) cells. Since transfected V5-tagged CLK induced both *per* and *tim* expression in S2 cells in our hands, we used RNAi against *per* and *tim* to eliminate any effect of PER and TIM proteins. After blocking protein synthesis with CHX, CLK showed robust degradation during the following 9 h (Figure 2C). When FLAG-HA-tagged CK2α was co-expressed, CLK degradation proceeded very slowly. The increase of CK2α levels by exogenous expression was rather limited in these conditions, indicating that a small increase in total CK2α protein can have substantial effects on CLK degradation. These results confirm the *in vivo* observations and strongly support a role for CK2α in the inhibition of CLK breakdown.

### CK2β Does Not Influence CLK Stability

**Figure 3:**
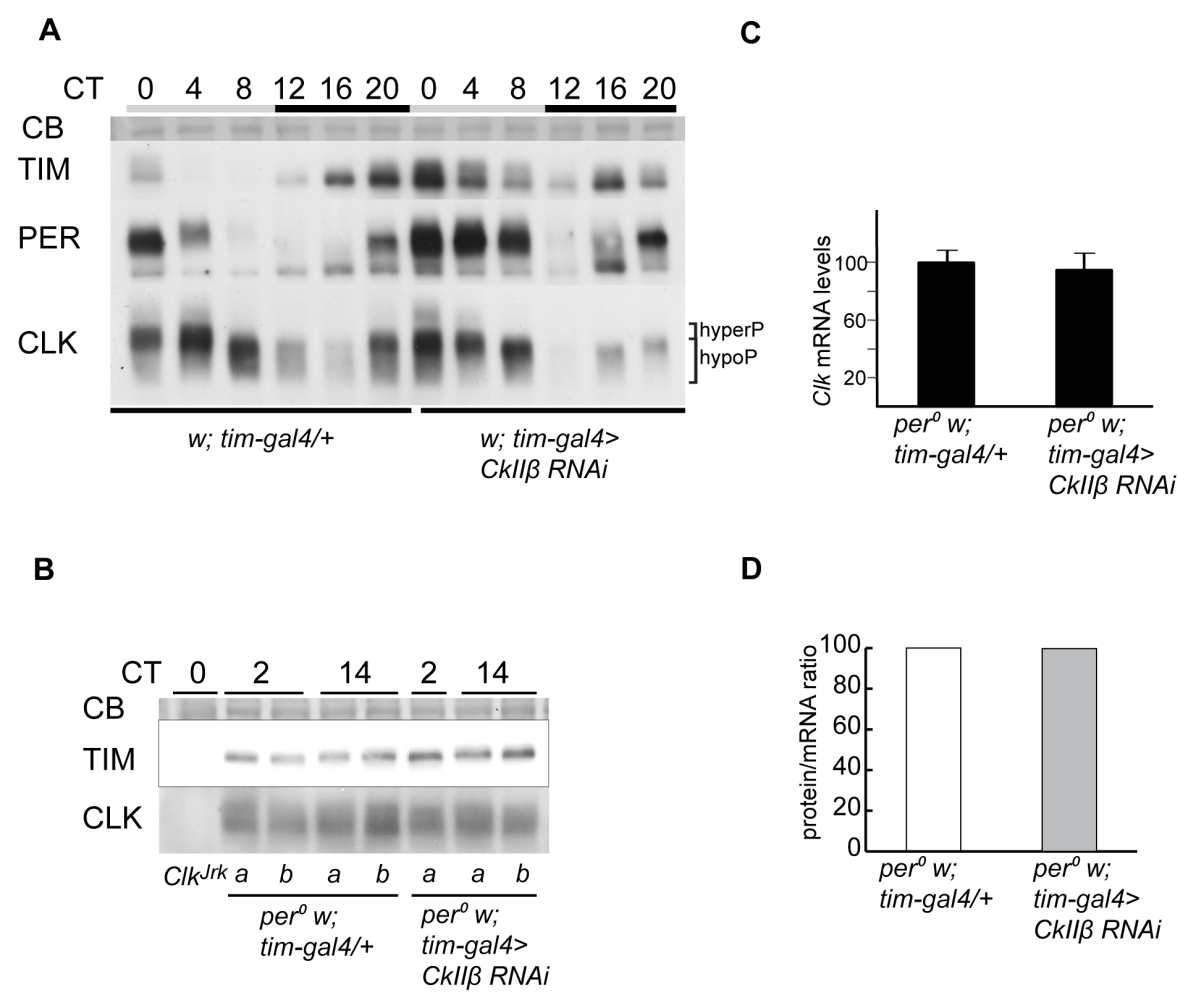
CK2β does not contribute to the inhibition of CLK degradation. (A, B) Western blot of nonsonicated head extracts from flies collected at DD1. Gray and black bars represent subjective day and subjective night, respectively. A CB stained band in the size range of CLK is used as a loading control. Brackets indicate hypo- and hyperphosphorylated forms of CLK. At least two independent experiments were performed for each blot. (A) Comparison between *tim > CkIIβ RNAi* (*w; tim-gal4/106845; 32377/+*) and *tim-gal4/+* controls in a *per^+^* background, for TIM, PER, and CLK proteins. (B) Comparison between *tim > CkIIβ RNAi* and *tim-gal4/+* controls in a *per^0^* background, for TIM and CLK. *a* and *b* are different protein extracts from the same genotype at the same time point. (C) Quantitative RT-PCR measurements of *Clk* mRNA levels in heads of flies collected at DD1. Results shown are means of pooled values from two time points (CT2 and 14, which gave similar values) with two independent samples for each time point. Error bars indicate s.e.m. Average values were normalized to the mean control (*per^0^ w*; *tim-gal4*/+) values set to 100. (D) CLK protein/*Clk* mRNA ratio calculated from mean CT2–CT14 values of Western blot (B) and quantitative RT-PCR (C) data. Ratios were normalized to the control (*per^0^ w*; *tim-gal4*/+) ratios set to 100.

Since inhibition of CK2α affected CLK stability and phosphorylation, we asked whether CK2β knockdown would have similar effects. *Pdf-gal4 UAS-CkIIβ-RNAi/+* flies have been reported to display long period rhythms [45]. Driving two *CkIIβ-RNAi* transgenes under the control of *tim-gal4* (hereafter *tim>CkIIβ-RNAi* flies, see Materials and Methods) induced behavioral arrhythmicity (Table 1). The specificity of the *CkIIβ* RNAi was first behaviorally assessed by rescue experiments involving *CkIIβ* RNAi under the control of the strong PDF^+^ cell driver *gal1118* [46] and the co-expression of different CK2β isoforms. The strongly altered behavior of *w;; gal1118/UAS-CkIIβ-RNAi* could be rescued by overexpression of the VIIa, VIIb, and VIIc CK2β isoforms (see [47]) (Table 1). Western blots against CK2β revealed a reduction in two isoforms in *tim>CkIIβ-RNAi* animals, while a third isoform remained unaffected (Figure S3A). TIM and PER cycling was profoundly altered in head extracts of *tim>CkIIβ-RNAi* flies at DD1 (Figures 3A and S3B). In contrast, CLK oscillations were only slightly affected. In particular, *tim>CkIIβ-RNAi* flies did not show the pronounced decrease in CLK levels that was observed in *tim>Tik* flies. Furthermore, CK2β depletion in a *per^0^* background did not result in a marked reduction of CLK phosphorylation or quantity (Figure 3B). Since equivalent levels of *Clk* mRNA were observed in *per^0^* flies with or without *CkIIβ* RNAi expression, their protein/mRNA ratios were identical (Figure 3C and D), in contrast to *tim>Tik* flies. Similarly, CLK was not affected when *CkIIβ* RNAi was expressed in a *tim^0^* background (not shown). In conclusion, although CK2α and CK2β proteins similarly affect TIM and PER accumulation and phosphorylation, the CK2β subunit does not seem to be required for CK2α to control CLK degradation and phosphorylation.

### CK2α Preferentially Forms Complexes with Highly Phosphorylated CLK in the Morning

**Figure 4:**
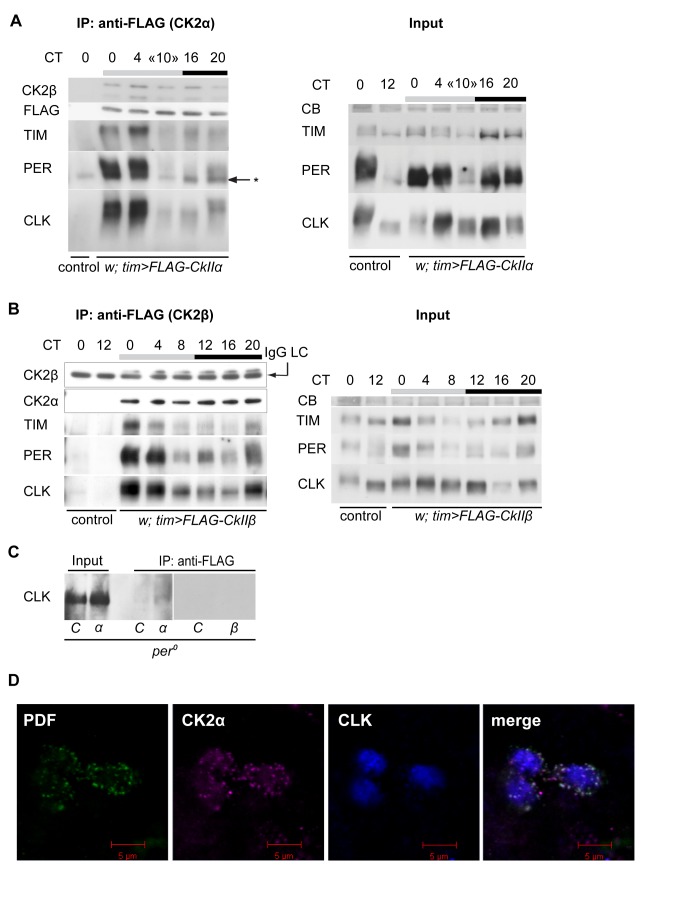
PER, TIM, and CLK are found in protein complexes containing CK2. Anti-FLAG immunoprecipitation (IP) from nonsonicated head extracts of flies collected at DD1. FLAG-CK2α or FLAG-CK2β was immunoprecipitated from 1 mg of total protein extracts from heads and 50% of the precipitate was subjected to Western blot analysis. We loaded 50 µg of head extracts as input controls. IgG LC indicates the immunoglobulin G light chain used for the precipitation that is well detected by the anti-mouse secondary antibody. Gray and black bars represent subjective day and subjective night, respectively. Experiments were performed at least twice. (A, Left) FLAG-CK2α was immunoprecipitated from *tim >FLAG-CkIIα* flies and *tim-gal4*/+ negative controls (control) in a *per^+^* background. IP of CK2α (FLAG) and co-IP of CK2β, TIM, PER, and CLK were visualized by immunoblotting. «*» shows an aspecific band recognized by the anti-PER antibody. (Right) TIM, PER, and CLK immunoblots of the corresponding inputs on *per^+^* background. The time “CT10” indicates a mixed population of flies harvested at CT8 and CT12. A CB stained band in the size range of CLK is used as a loading control. TIM was run on a 3–8% Tris-Acetate gel. (B, Left) FLAG-CK2β was immunoprecipitated from *tim > FLAG-CkIIß* flies and *tim-gal4*/+ negative controls (control) in a *per^+^* background. IP of CK2ß (with anti-CK2ß) and co-IP of CK2α, TIM, PER, and CLK were visualized by immunoblotting. (Right) TIM, PER, and CLK immunoblots of the corresponding inputs. A CB stained band in the size range of CLK is used as a loading control. (C) FLAG-CK2α or FLAG-CK2β was immunoprecipitated from *tim >FLAG-CkIIα* (*α*) flies and *tim-gal4*/+ negative controls (C) in a *per^0^* background. co-IP of CLK was visualized by immunoblotting. Input samples and immunoprecipitates were run on the same gel for *C* and *α.* (D) Image showing PDF (green), CK2α (magenta), and CLK (blue) fluorescent immunolabeling in small PDF^+^ LNv-s of an adult fly at ZT3. The fourth square is a composite picture of the three stainings. Single optical planes are shown taken by confocal microscopy.

The strong effects of CK2α inhibition on CLK suggested that the two proteins might physically interact. Flies expressing a FLAG-tagged CK2α protein under *tim-gal4* control displayed strong behavioral rhythms with a 1 h period lengthening (Table 1). Anti-FLAG immunoprecipitation experiments were performed from FLAG-CK2α-expressing fly head extracts at different circadian times and showed co-immunoprecipitation of TIM, PER, and CLK mostly at the end of the subjective night and in the subjective morning when these proteins are mainly hyperphosphorylated (Figure 4A). Although relatively abundant medium-phosphorylated clock proteins were observed in the extracts at CT16, they were poorly co-immunoprecipitated with CK2α. The CK2α subunit thus appears to preferentially make complexes with highly phosphorylated forms of TIM, PER, and CLK. Flies expressing FLAG-tagged CK2β were also behaviorally rhythmic with a slightly lengthened period (Table 1), and FLAG-CK2β expression could rescue the severe period lengthening induced by *CkIIβ* RNAi (Table 1), indicating that the tagged protein was functional. Similarly to CK2α, CK2β was found to be associated with hyperphosphorylated TIM, PER, and CLK in the late subjective night and in the subjective morning, whereas little amounts of proteins were co-immunoprecipitated at other circadian times (Figure 4B). The results thus suggest that CK2 holoenzyme is involved in the hyperphosphorylation of CLK, PER, and TIM in the late night/morning part of the cycle.

Since CK2α strongly influences CLK stability in the absence of PER, anti-FLAG immunoprecipitations were also done in *per^0^ tim>FLAG-CkIIα* flies (Figure 4C). Minute amounts of hypophosphorylated CLK were co-immunoprecipitated in *per^0^* extracts, nevertheless indicating that CLK-CK2α complexes may exist in the absence of PER. Conversely, CK2β did not co-immunoprecipitate with CLK in a *per^0^* background (Figure 4C). The poor detection of CK2α–CLK complexes in the absence of PER suggested a very labile interaction between the two proteins or indirect PER-independent effects of CK2α on CLK.

CK2 subunits preferentially associate with clock proteins at times when those are present in the nucleus. CK2α was, however, described to localize to the cytoplasm of LNv-s [8]. We therefore set out to investigate whether CK2α could be present in the nucleus of LNv-s as well. Whole-mount adult brains were stained with an anti-CK2α antibody together with anti-PDF and anti-CLK. PDF is known to be exclusively cytoplasmic [48], while CLK is almost completely nuclear in our hands (see also [26]). Although CK2α predominantly localized to the cytoplasm of s-LNv-s, a fine cloud of CK2α staining co-localized with CLK to the nucleus (Figure 4D).

### CK2α Increases CLK Phosphorylation in a PER-Dependent Manner

**Figure 5:**
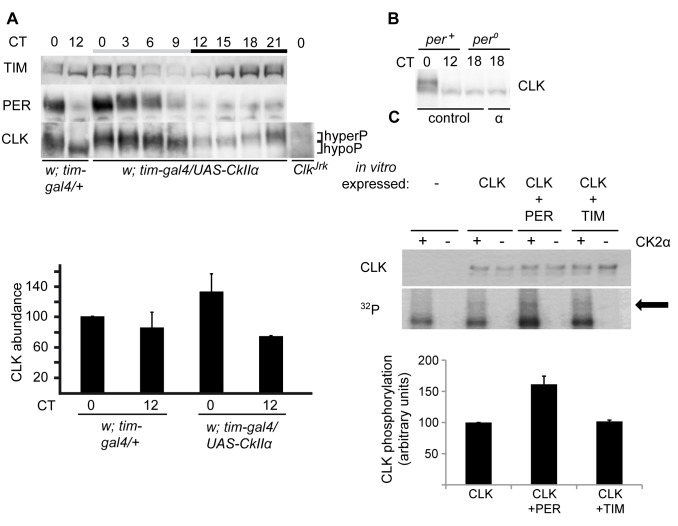
CK2α overexpression induces CLK hyperphosphorylation in the presence of PER. (A, B) Western blot of nonsonicated head extracts from flies collected at DD1. Samples were run on 3–8% Tris-Acetate gels in order to better resolve hyperphosphorylated CLK and TIM forms. Gray and black bars represent subjective day and subjective night, respectively. At least two independent experiments were performed for each blot. (A, Top) Comparison between *tim > CkIIα* and *tim-gal4/+* controls in a *per^+^* background, for TIM, PER, and CLK proteins. (Bottom) Two independent experiments as above were quantified for CLK abundance, and the mean values are plotted. Error bars stand for the difference of the respective values from each experiment and their mean. The value of *w; tim-gal4/+*at CT0 was normalized to 100. (B) Comparison between *per^0^*; *tim > CkIIα* and *per^0^*; *tim-gal4/+* controls for CLK. (C) CK2α phosphorylates CLK *in vitro*. (Top) Wild-type CLK was translated with a N-terminal 6-histidine fusion tag *in vitro*, affinity purified either in the absence or presence of PER or TIM, and subjected to phosphorylation assays by incubation with γ–^32^P-ATP either in the absence (−) or presence (+) of CK2α. Intensity of incorporated ^32^P-phosphate into CLK (^32^P) was analyzed by autoradiography and total CLK protein levels (CLK) were determined by Western blot analysis. The arrow indicates the position of phosphorylated CLK. (Bottom) Quantification of CLK-incorporated ^32^P-phosphate after normalization towards total CLK protein levels. Average CLK phosphorylation from at least three experiments ± s.e.m. are shown in the figure with wild-type CLK set to 100.

To further decipher the function of CK2α in CLK phosphorylation, CLK protein was analyzed in flies overexpressing wild-type CK2α. As previously reported [41], CK2α overexpression induced a modest lengthening of the behavioral period (Table 1). *w;tim-gal4/UAS-CkIIα* (*tim>CkIIα*) flies showed subtle changes of PER and TIM oscillations with a slightly delayed degradation of the TIM (CT 4–8) and PER (CT 8) proteins during daytime at DD1 (Figures 5A and S4A–B). CLK levels in CK2α overexpressing head extracts were higher at CT0 and lower at CT12 compared to controls, but overall protein levels were not significantly affected (Figure 5A). In contrast, CLK phosphorylation was strongly altered, with forms always more phosphorylated than the wild-type minimal phosphorylation that is observed at CT12 (Figure 5A). CLK phosphorylation was not increased by CK2α overexpression in a *per^0^* background (Figure 5B), indicating that CLK hyperphosphorylation by CK2α required PER. The results thus support a PER-dependent hyperphosphorylation of CLK by CK2α, whereas CLK hypophosphorylation and stability appears to be mostly controlled by a PER-independent CK2α function.

The CLK phosphorylation defects in flies with altered CK2α functions and the presence of CLK-CK2α/β complexes suggested that CK2 might directly phosphorylate CLK. We thus asked whether the CK2 holoenzyme could phosphorylate CLK *in vitro*. Indeed, CLK was phosphorylated by CK2, and the presence of PER increased CLK phosphorylation by about twofold (Figure S4C). Addition of TIM protein did not affect the CK2-dependent phosphorylation of CLK. When only the CK2α catalytic subunit was used for the *in vitro* assay, CLK was phosphorylated with a similar efficiency and showed the same PER-mediated facilitation of its phosphorylation (Figure 5C). This confirms the *in vivo* results indicating that at least some of the CK2α effects on CLK phosphorylation do not require CK2β, and supports a direct phosphorylation of CLK by CK2α.

### CK2α Decreases CLK Transcriptional Activity

**Figure 6:**
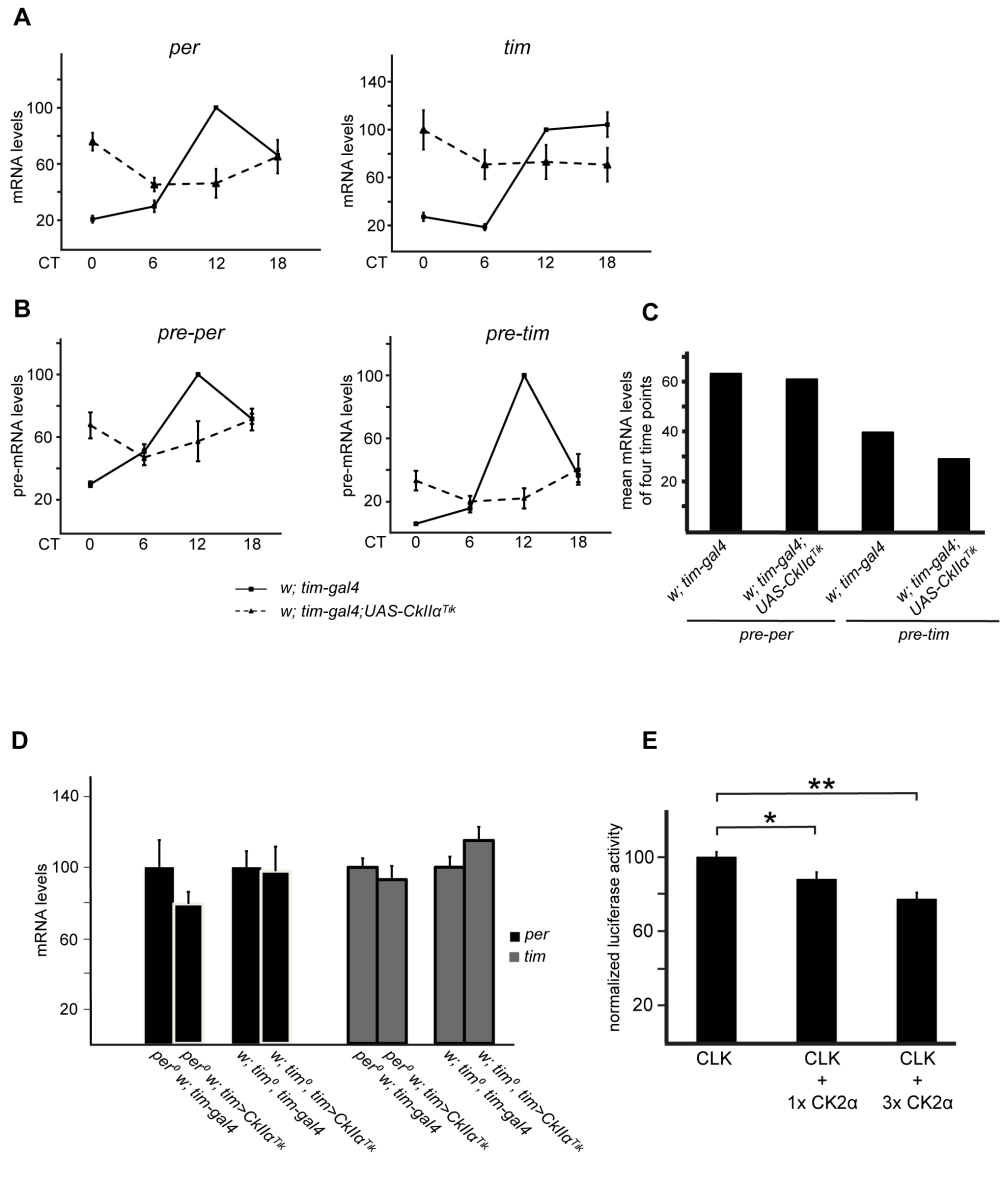
CK2α decreases CLK transcription factor activity. (A–D) Quantitative RT-PCR measurements of *per*, pre*–per*, *tim*, and pre–*tim* mRNA levels in heads of flies collected at DD1. Error bars indicate s.e.m. (A) *per* and *tim* mRNA levels in *tim>Tik* and control flies. Values were normalized to the maximum value (control at CT12) set to 100. Mean mRNA levels +/− s.e.m. from at least three independent experiments are shown. (B) Quantitative RT-PCR measurements of *per and tim* pre-mRNA levels in heads of *tim >Tik* and control flies collected at DD1. Average values from three independent experiments were normalized to the control (*w; tim-gal4*) mean value at CT12 set to 100. Error bars represent s.e.m. (C) Relative qPCR values from Figure 6B were averaged from the four indicated time points (CT0, CT6, CT12, and CT18) for each genotype and for each pre-messenger and the mean value was plotted. 100 stands for the highest pre-mRNA expression in the respective genotype at CT12. (D) *per* and *tim* mRNA levels in *tim > Tik* and controls in a *per^0^* or *tim^0^* background. Results are means of pooled values from two time points (CT2 and 14). Values were normalized to the corresponding controls set to 100. Previous analysis of separate values at CT2 and CT14 indicated that they were similar (Table S1), justifying their common treatment (see above). Average results from at least three independent experiments are shown. (E) Luciferase activity assay in the presence or absence of CK2α overexpression. S2 cells were transfected, harvested, and measured as described in Materials and Methods. We transfected 5 ng pAc-*Clk*-V5, 10 ng p3x69-luc, and 10 ng pAc-Renilla luciferase with or without 5 or 15 ng of the FMO02931 CK2α expression vector. Mean luciferase activity +/− s.e.m. of at least four different samples from two independent experiments are shown. CLK-dependent luciferase expression in the absence of CK2α co-expression was set to 100. 1× CK2α indicates 5 ng FMO02931, and 3× CK2α stands for 15 ng FMO02931. Student's *t* test (unpaired, two-tailed) was applied to the respective groups, * *p* = 0.0315, ** *p* = 0.00118.

The strong influence of CK2α on CLK phosphorylation and stability suggests that CLK-dependent transcription could be affected in flies with altered CK2α activity. As previously reported [42], intermediate levels of *per* and *tim* mRNAs were observed in *tim>Tik* flies (Figure 6A).

*per* and *tim* pre-mRNA levels were measured to more directly estimate CLK transcriptional activity. Average nonoscillating levels of *pre*–*per* and *pre*–*tim* were observed in *tim>Tik* flies (Figure 6B,C), despite the very reduced amounts of CLK protein (see Figure 1A). It thus suggested that CLK transcriptional activity was strongly increased when CK2α activity was diminished. Flies expressing the CK2α^Tik^ protein in a *per^0^*, *tim^0^*, or *per^0^ tim^0^* double mutant background revealed no statistically significant changes in *per* and *tim* mRNA levels compared to wild-type CK2α controls (Figures 6D and S5A). However, CLK protein levels were reduced to 25–50% in CK2α^Tik^ expressing flies (see Figures 2 and S2), suggesting that specific CLK activity was still increased. Effects of CK2α on the transcriptional activity of CLK thus appears to be at least partly independent of PER and TIM. *per* and *tim* mRNA levels were also measured in *tim>CkIIβ-RNAi* flies and showed intermediate levels compared to controls (Figure S5B). Since CLK quantity is unaffected by *tim>CkIIβ-RNAi* (Figure 3A), CK2ß does not strongly modify CLK transcriptional activity.

Finally, CLK-dependent transcription was tested by CLK-induced reporter gene expression in S2 cells. On a CLK-binding synthetic minimal enhancer composed of three *per*-derived E-boxes, CLK-dependent transcription was decreased in a dose-dependent manner by CK2α co-expression (Figure 6E). Since CLK quantity was increased in the presence of CK2α overexpression (see Figure 2C), the transcriptional decrease could hardly be a consequence of lower CLK levels.

## Discussion

Temporally controlled phosphorylation of clock proteins is a key feature of the transcriptional-translational negative feedback loop underlying the *Drosophila* circadian clock. Although the CLK activator shows robust oscillations of its phosphorylation levels, the phosphorylation mechanisms and how they affect CLK function remain largely unknown. Our study aimed at determining whether CK2 was involved in the control of CLK phosphorylation and how it would affect CLK circadian function. Overexpression of the CK2α^Tik^ dominant-negative enzyme or RNA interference against *CkIIα* substantially reduced CLK phosphorylation as well as protein levels. In accordance with the *in vivo* observations, co-transfection of CK2α with CLK in S2 cells increased CLK stability. This supports a function for CK2α in CLK stabilization. High CLK target gene transcription was induced by the CK2α^Tik^ protein despite the low-level accumulation of CLK, indicating that CK2α decreased the expression of CLK targets. This was further corroborated in the luciferase activity assay in S2 cells where overexpression of CK2α inhibited CLK activity. Furthermore, CK2α associated with hyperphosphorylated forms of CLK in the morning and it was able to directly phosphorylate the CLK protein *in vitro*. Effects of CK2α on CLK stability and activity did not require PER or TIM, but CLK phosphorylation by CK2α involves both PER-independent and PER-dependent functions. The results suggest that direct phosphorylation by CK2α stabilizes CLK and diminishes its transcriptional activity.

CLK protein levels but not *Clk* mRNA levels are low in *tim>Tik* and *tim> CkIIα-RNAi* flies, indicating that CK2 specifically affects CLK protein levels. Although a role of CK2α in CLK protein synthesis cannot be completely excluded, three sets of experimental data support a posttranslational action of CK2α on CLK. First, CK2α associates with CLK, PER, and TIM in protein complexes. Second, CK2α affects CLK phosphorylation state *in vivo* and is able to phosphorylate CLK directly *in vitro*. Third, CK2α stabilizes CLK even after protein synthesis blockage with CHX. Importantly, *tim>Tik* flies show reduced CLK phosphorylation, as predicted from kinase inhibition. This is in contrast with the effects of *CkIIα^Tik^* on PER and TIM, for which highly phosphorylated forms of the proteins accumulate, although PER phosphorylation remains lower than the highest state in the wild-type [8],[15],[41],[42]. Nevertheless, CK2 is able to phosphorylate PER and TIM *in vitro* [8],[41],[49].

The *CkIIα^Tik^* mutation affects TIM phosphorylation in the absence of PER, whereas TIM is required to observe effects of *CkIIα^Tik^* on PER [15]. TIM was thus proposed to be a direct target of CK2 that drives CK2-dependent modification of PER [15]. One might expect that TIM or PER relays the effects of CK2 on CLK. This is not supported by the strong effect of *CkIIα^Tik^* on the CLK protein in *per^01^*, *tim^01^*, or *per^01^ tim^01^* double mutants. However, PER strongly influences CLK phosphorylation by CK2. First, overexpression of wild-type CK2α induces CLK hyperphosphorylation in *per+* but not in *per^0^* flies. Second, PER enhances *in vitro* phosphorylation of CLK by CK2α. Finally, the abundance of CLK/CK2 complexes observed in head extracts is much lower in *per^0^* mutants. In comparison to *per^0^*, wild-type flies accumulate much more CLK/CK2α complexes, particularly in the morning when PER and CLK are abundant and hyperphosphorylated. The PER–CLK interaction is the strongest in the morning and the weakest in the early evening when PER is highly degraded. It seems unlikely that this temporal pattern of CLK interactions with PER is strongly altered in FLAG-CK2α overexpressing animals used for the immunoprecipitation since they show behavioral and molecular rhythms similar to wild-type flies. CLK/CK2α complexes strongly decrease after CT4 when high levels of CLK but not PER remain, suggesting that CLK/CK2α interactions follow phosphorylated PER abundance. PER hence could drive a large fraction of CLK/CK2α interactions with PER-free CLK being a weaker CK2 substrate. Since PER interacts in the late night with CLK species that no longer bind chromatin [22], it suggests that CLK/PER/CK2α complexes are mostly unbound to DNA.

PER/DBT-dependent phosphorylation marks CLK for degradation [19],[20]. Although the NEMO kinase destabilizes CLK [29], whether it acts as a PER/DBT-dependent CLK kinase is not known. Our results indicate that inhibiting CK2α activity increases CLK breakdown, whereas overexpressing CK2α induces accumulation of highly phosphorylated CLK. CK2α thus appears to have opposite effects on CLK stability, compared to DBT and NEMO. Since both CK1 (DBT) and CK2 show a preferential association with CLK in the morning, they might counteract each other to control CLK degradation and recycling for a next transcription cycle. Interestingly, a kinase complex that includes CK1 promotes the SUPERNUMERARY LIMBS (SLMB)-dependent proteolysis of the CUBITUS INTERRUPTUS (CI) transcription factor, whereas CK2 stabilizes CI by preventing its ubiquitylation [50],[51].

As opposed to *tim>Tik* flies, flies expressing *CkIIβ* RNAi did not show significantly decreased CLK levels. In addition, CK2α and the CK2 holoenzyme are both able to phosphorylate *Drosophila* CLK *in vitro*. Our data suggest that CLK, in contrast to PER and TIM, might be a substrate of CK2α alone rather than a substrate of the CK2 holoenzyme *in vivo*. Several studies suggest that CK2α and β do not act synergistically on a handful of substrates [52] or even play antagonist roles with CK2β inhibiting CK2α-dependent phosphorylation of some target proteins (see [40]). In mammals, CK2α is more efficient than the CK2 holoenzyme to phosphorylate BMAL1, and can also phosphorylate CLK [53].

As previously reported [15],[42], *tim>Tik* flies showed intermediate levels of *per* and *tim* transcripts. We corroborated the involvement of transcription in this phenomenon by determining *per* and *tim* pre-mRNA profiles. It has been proposed that the high levels of hyperphosphorylated TIM in *tim>Tik* would prevent normal PER-dependent transcriptional repression [15]. However, the fact that flies expressing the CK2α^Tik^ protein in a *per^0^*, *tim^0^*, or *per^0^ tim^0^* double mutant background have only half dose (or less) of CLK but as high levels of *per* and *tim* transcripts as the *CkIIα* ^+^ controls supports an additional PER/TIM-independent transcriptional function of CK2. Importantly, the small amount of remaining CLK protein in the late night in *per^+^ tim>Tik* flies drives similarly high pre*–per* expression as much more CLK in the wild-type (see Figure 6B). That also undermines CK2's involvement only in PER/TIM repressor function during CLK-mediated transcription. A likely explanation is that the low-level hypophosphorylated CLK is extremely active in flies with reduced CK2α activity. In line with the *in vivo* results, the luciferase activity assay in cultured S2 cells uncovered a dose-dependent repression of CLK activity by the CK2α subunit on a minimal enhancer-promoter element.

CK2α thus appears to control CLK-dependent transcription by increasing PER/TIM repressing capacity and jointly decreasing CLK activity by some other mechanism. CK2β supports TIM-dependent repression (Figure S5B), but may not contribute to the PER/TIM-independent control of CLK activity by CK2α. Since deubiquitylation of CLK by USP8 decreases its activity [25], it will be interesting to investigate whether CK2α phosphorylation affects CLK ubiquitylation.

In the *Neurospora* circadian transcriptional feedback loop, the FRQ repressor recruits CK1 and CK2 to promote phosphorylation of the WCC activator complex resulting in the inhibition of its transcriptional activity [35],[54]–[57]. Reactivation of WCC occurs through its dephosphorylation by phosphatases such as PP2A [54],[56]. WCC is destabilized when turning active and gets stabilized as soon as it resumes a transcriptionally inactive state [56],[57]. This is reminiscent of our finding about the role of CK2 in CLK activity regulation. Recently, BMAL1 and CLK were also shown to be “Kamikaze” activators in mammals in that their activity was dependent on proteasome function—highly unstable CLK and BMAL1 were the most active, while proteasome inhibition resulted in long-lived but less potent activators [31],[32],[58]. Our findings indicate that CK2 might be a key player in such a mechanism, by promoting CLK stability and decreasing its activity. It remains to be seen how CK2 and DBT-dependent kinase activities interact on CLK to set CLK transcriptional activity to a proper phase in the circadian cycle.

## Materials and Methods

### Fly Stocks and Constructs

*Drosophila melanogaster* stocks were maintained on a 12 h∶12 h LD cycle on standard corn meal-yeast-agar medium at 25°C. *Clk^Jrk^* is a dominant allele of *Clk*, which results in a truncated and highly unstable CLK protein [59]. *per^01^* [60], *tim^01^* [61], *w;tim-gal4-62* [62], *w;;gal1118* [46], *per^01^w*;;*13.2(per(Δ)-HA10His) F21* [63], *yw;;P{UAS-CkIIα.Tik} T1* [15], *yw;P{UAS-CkIIα.L} 35* [41], *w;UAS-FLAG-CkIIα* [51], and lines carrying UAS transgenes encoding each of the five CK2β isoforms [64] have been previously described. The *gal1118* driver line in the adult brain is expressed in the small and large LNv-s in addition to some few nonclock cells [46]. *UAS-RNAi* flies against *CkIIβ* (stocks 32377 and 106845) and *CkIIα* (stock 17520 R-2) are described in http://stockcenter.vdrc.at/control/main and http://www.shigen.nig.ac.jp/fly/nigfly/index.jsp, respectively. Both *CkIIβ* RNAi lines (32377 and 106845) were induced in all the experiments using *CkIIβ* RNAi except specifically indicated. The *UAS-FLAG-CkIIβ* construct was made by inserting a FLAG-CK2ß coding segment (kindly provided by A. Bidwai, West Virginia University) into the pUAST vector, and *w;UAS-FLAG*-*CkIIβ* transgenic flies were generated by standard procedures. For *in vitro* phosphorylation assays, *Clk* constructs with a 6-histidine fusion tag as well as *per* and *tim* were expressed from a SP6 promoter incorporated in a pAc-5.1 vector, as described previously [30]. The FMO02931 expression plasmid was obtained from the Drosophila Genomics Resource Center (DGRC). It contains the full *CkIIα* ORF tagged C-terminally with FLAG and HA and driven by the metallothionein promoter. We verified the *CkIIα* ORF and the promoter region by sequencing.

### Behavioral Analysis

Behavioral assays for locomotor activity rhythms were carried out with 1- to 5-d-old males at 25°C in *Drosophila* activity monitors (TriKinetics). Illumination was provided by standard white fluorescent low-energy bulbs. Light intensity at fly level was in the range of 300–1000 µW/cm^2^. Flies were first entrained to 12 h∶12 h light-dark (LD) cycles for 4 d and then transferred to constant darkness (DD). Activity data were analyzed from the second to the ninth day in DD. Data analysis was done with the FaasX 1.16 software that is derived from the Brandeis Rhythm Package (see [65]) and is freely available upon request (Apple Mac OSX only). Rhythmic flies were defined by χ2 periodogram analysis of an 8-d dataset with the following criteria (filter ON): power ≥20, width ≥1.5 h, with no selection on period value. Power and width represent the height and width of the periodogram peak, respectively, and give the significance of the calculated period. Genotypes with a reduced number of rhythmic flies (<50%), low power (<50), and high s.e.m. of the period (>1) are considered arrhythmic. Experiments were reproduced two or three times with very similar results.

### Protein Sample Preparation, Phosphatase Treatment, Sonication, and Western Blotting

We entrained 1 to 5-d-old flies to 12 h∶12 h LD cycles for 4 d and transferred to DD (CT0 is 12 h after the last lights-OFF). Flies were collected on dry ice during the first day of DD (CT0–24). We homogenized 30–60 heads on ice in a modified RBS buffer [20]: 10 mM HEPES pH 7.5, 5 mM Tris-HCl pH 7.5, 50 mM KCl, 10% glycerol, 2 mM EDTA, 1% Triton X-100, 0.4% NP-40, 1 mM DTT, Complete Mini Protease Inhibitor Cocktail Tablet (Roche), Phosphatase Inhibitor Cocktail 2 and 3 (Sigma-Aldrich), and 20 mM β-glycerophosphate (3–4 µl buffer/head). A Brinkmann Heidolph Mechanical Overhead Stirrer RZR1 was used for the homogenization. After 1 min of extraction, tubes were incubated in ice for 30 min, then homogenized again for another minute. If sonication was included after this step, samples were sonicated on ice with a Vibracell ultrasonic processor (Bioblock Scientific) at 4W output for 5×10 s with 1 s breaks. Following Bradford protein concentration measurement (BioRad), supernatants were used for polyacrylamide gel electrophoresis. When supernatants were treated with λ protein phosphatase, 1,600 units of λ protein phosphatase (New England Biolabs) and 1 mM MnCl_2_ were added to sonicated extracts prepared in phosphatase inhibitor-free buffer [10 mM HEPES pH 7.5, 100 mM KCl, 0.1 mM EDTA, 5% glycerol, 0.1% Triton X-100, 5 mM DTT, and EDTA-free Complete Mini Protease Inhibitor Cocktail Tablet (Roche)] and subsequently incubated for 30 min at 30°C. Reaction was stopped by adding 1× NuPAGE LDS sample buffer (Life Technologies), 500 mM DTT, and incubation for 10 min at 70°C. We loaded 50 µg total protein on Novex 4% Tris-Glycine precast gels (Life Technologies) for PER, TIM, and CLK immunoblotting, except specifically indicated. When indicated, NuPAGE Novex 3–8% Tris-Acetate gels were used for TIM and CLK immunoblotting for a better resolution of hyperphosphorylated forms. Samples (50 µg) for FLAG, CK2α, and CK2β immunoblots were run on NuPAGE Novex 4–12% Bis-Tris precast gels (Life Technologies). Electrophoresis and blotting were done according to the manufacturer's instructions except for a 3 h running time for Tris-Acetate and a 2 h running time for Tris-Glycine gels. Equal loading was verified by Ponceau S staining on blotting membranes, which were blocked in 5% nonfat dry milk in TBST (Tris-Buffered Saline with 0.1% Tween-20) for 1 h at 25°C and then incubated with the primary antibody overnight at 4°C. The following primary antibodies were used diluted in 5% milk in TBST: rabbit anti-V5 (Sigma-Aldrich V8137, Lot 019K4827) at 1∶4,000, rabbit anti-myosin heavy chain (MHC, kind gift of Roger E. Karess, Institut Jacques Monod, Paris) at 1∶400,000, rabbit anti-CK2α (Abcam ab81435) at 1∶1,000, mouse anti-CK2β (Calbiochem 6D5 218712) at 1∶1,000, rat anti-TIM [7] at 1∶2,000, goat anti-CLK (Santa Cruz Biotechnology sc27070) at 1∶1000, rabbit anti-PER [66] at 1∶10,000, guinea pig anti-VRILLE [44] at 1∶5,000, and guinea pig anti-PDP1ε [67] at 1∶5,000. For immunoblotting of anti-FLAG immunoprecipitations, guinea pig GP90 anti-CLK [18] at 1∶1,000 was used since an aspecific IgG-derived band revealed with the SC27070 anti-CLK on the immunoprecipitates. Membranes were washed three times for 10 min, then the HRP-conjugated secondary antibodies (Santa Cruz Biotechnology) were added diluted in 5% milk in TBST: goat anti-rabbit (1∶10,000), goat anti-rat (1∶20,000), goat anti-mouse (1∶20,000), donkey anti-goat (1∶10,000), and goat anti-guinea pig (1∶10,000). In the case of anti-CK2β, TrueBlot ULTRA Anti-Mouse IgG-HRP (eBioscience, 1∶2,000) was used as a secondary antibody to circumvent problems resulting from primary antibody light chain detection after immunoprecipitation.

Blots were revealed with the Amersham ECL Plus reagent (GE Healthcare). SimplyBlue SafeStain (Life Technologies) was used to stain membranes after blotting. Images were quantified with the NIH ImageJ (1.43 k) software after background subtraction. Calculations were done and histograms were generated with Microsoft Excel.

### Immunoprecipitation

Fly head extracts were prepared as described above, except that HE buffer [20 mM HEPES pH 7.5, 150 mM KCl, 0.1 mM EDTA, 0.1% NP-40, 5% glycerol, Complete Mini Protease Inhibitor Cocktail Tablet (Roche), Phosphatase Inhibitor Cocktail 2 and 3 (Sigma-Aldrich) and 20 mM β-glycerophosphate] was used for the homogenization. We incubated 1 or 2 mg total protein overnight at 4°C with either 25 µl EZview Red anti-FLAG M2 Affinity Gel (Sigma-Aldrich) or 25 µl Protein G Sepharose (Pierce) mixed with 10 µl of anti-CLK antibody (Santa Cruz Biotechnology SC27069). Following three 10 min washes, bound complexes were eluted in 1× NuPAGE LDS sample buffer (Life Technologies) without DTT for 10 min at 70°C. Supernatants were complemented with DTT (500 mM) and reduced for 10 min at 70°C.

### Cell Culture Experiments

Drosophila Schneider 2 (S2) cells [kind gift of Anne Plessis (Institut Jaques Monod, Paris)] were maintained in SFX-Insect Medium (HyClone) supplemented with 10% fetal bovine serum (Sigma-Aldrich) and 1% penicillin-streptomycin solution (Sigma-Aldrich) as previously described [68]. Complementary single-stranded RNA-s were *in vitro* transcribed from purified PCR templates containing the T7 RNA polymerase promoter site on both ends, using the MEGAscript T7 Kit (Life Technologies). Reactions were purified with the MEGAclear Kit (Life Technologies) and precipitated in ethanol/sodium acetate for concentration followed by resuspension in 40 µl H_2_O and annealing of the two strands (30 min at 65°C and slow cooling to room temperature). RNA quality and quantity was assessed by spectrophotometry and agarose gel electrophoresis. Primers for *per* target sequence amplification by PCR were: TTAATACGACTCACTATAGGGAGAAAGGAGGACAGCTTCTGCTGC and TTAATACGACTCACTATAGGGAGAGATATGATCCCGGTGGCCGTG and for *tim* were: TTAATA; CGACTCACTATAGGGAGACTGGTTACTAGCAACTCCGCA and TTAATACGACTCA; and CTATAGGGAGAGCAGGATATTTCTCAGCAGCA.

pAc-*Clk*-V5/His6 [10], pAc-Renilla luciferase (kind gift of M. Rosbash), and p3x69-luc (containing three copies of *per* E-box as enhancer element [10],[69]) were already described. Transient transfection was performed with Effectene (Qiagen) using plasmids purified with the Plasmid Midi Kit (Qiagen). DNA quantities were equalized for transfection by addition of empty pAc vector. The induction of *CkIIα* under the control of metallothionein promoter was achieved by adding 500 µM CuSO_4_ to the cells 1 d after transfection.

For luciferase activity assays, 10^6^ cells were seeded in six-well plates, left to proliferate in serum-free medium for 48 h, were transfected in serum-free medium, supplemented with serum and antibiotics 4 h later, and harvested 48 h posttransfection. Cells were washed in PBS and lysed on plate with Passive Lysis Buffer according to the Dual-Luciferase Reporter Assay System manual (Promega). Lysates were cleared by centrifugation at 4°C and 10 µl of supernatant was measured for firefly and Renilla luciferase activities with the Dual-Luciferase Reporter Assay System (Promega) on a Mithras LB 940 luminometer (Berthold Technologies). Firefly luciferase activities were normalized to corresponding Renilla luciferase activities to control for transfection efficiency and protein concentration. Experiments were made in duplicates or quadruplicates and repeated at least twice.

For degradation assays, cells were seeded in 60 mm dishes (2.5×10^6^ cells/dish) and treated with *per* and *tim* dsRNA (37.5 µg) in serum-free medium for 48 h followed by transfections in medium containing serum and antibiotics. One day posttransfection, cells were split in four equal volumes and seeded in 12-well plates followed by induction with CuSO_4_. One day after induction, cycloheximide (CHX, Sigma-Aldrich) was added to each well at a final concentration of 0.58 mM, and cells were harvested 0, 3, 6, and 9 h after the beginning of CHX treatment. After harvest, cells were centrifuged for 5 min at 2,000 *g* at 20°C, washed once with PBS, and pellets were frozen at −80°C until extraction. Protein extraction was achieved by lysing cells in 40 µl of HE buffer (described above) supplemented with 0.5% Triton X-100 by means of pipetting and vortexing. After centrifugation for 10 min at 14,000 rpm at 4°C, supernatants were subjected to Bradford assay. We used 20 µg protein for polyacrylamide gel electrophoresis. Blots were revealed with anti-V5 for CLK and with anti-MHC as a loading control. Both blots were quantified by ImageJ. V5 reactivity was normalized to MHC reactivity for each sample, which was used for the calculations that are plotted in Figure 2E.

### *In Vitro* Phosphorylation Assays

*In vitro* transcription/translation and phosphorylation reactions were carried out as described previously [30], with the following differences: CLK protein with a N-terminal 6-histidin fusion tag as well as PER and TIM were expressed in TNT SP6-Quick Coupled High Yield Wheat Germ expression system (Promega) for 2 h at 25°C with the addition of 0.2 mM staurosporine to block phosphorylation. Subsequently CLK protein was precipitated with 20 µl nickel-nitrilotriacetate (Ni-NTA) agarose for 90 min at 4°C either with or without prior addition of PER or TIM expressing lysates. Affinity purified CLK protein with or without co-precipitated PER or TIM was subjected to on bead phosphorylation reactions by human casein kinase II holoenzyme (New England Biolabs) or recombinant human CK2α subunit (KinaseDetect, DK-5792 Aarslev, Denmark) in 50 µl phosphorylation buffer (20 mM Tris-HCl, pH 7.5, 50 mM KCl, 10 mM MgCl_2_) with 0.5 µCi/µl γ–^32^P-ATP at 30°C for the holoenzyme and at 37°C for CK2α. The amount of CLK-incorporated ^32^P-phosphate was quantified by autoradiography and densitometry after SDS-page electrophoresis and blotting to nitrocellulose membrane. The intensity of the ^32^P-signal was normalized by total CLK protein level, as quantified by Western blot analysis.

### Quantitative RT-PCR

Total RNA was prepared from adult heads (about 35) using the Promega SV Total RNA Isolation System. It was quantified using the Nanodrop ND-1000 spectrophotometer, and the integrity of the RNA was verified using the Agilent 2100 bioanalyser with the eukaryote total RNA Nano assay. RNA was treated with rDNase (NucleoSpin RNA Kit, Macherey-Nagel) in solution after RNA isolation to ensure optimal conditions for pre-mRNA detection. One µg of total RNA was reverse-transcribed in a 50 µl final reaction in presence of 0.4 µM oligodT(15) or random hexamer primers (for detection of pre-mRNA-s), 8 mM dNTP, 40 units of RNasine, and 400 units of M-MLV RTase H-minus (Promega), during 3 h at 37°C. Quantitative PCR was performed with a Roche LightCycler (mRNA-s) or an Applied Biosystems 7900HT Fast Real-Time PCR System (pre-mRNA-s) using the SYBR green detection protocol of the manufacturer. We mixed 3 µl of a 25× diluted cDNA (or 1 ng/µl) with FastStart DNA MasterPLUS SYBR green I mix with 500 nM of each primer, and the reaction mix was loaded on the capillaries and submitted to 40 cycles of PCR (95°C/15 s; 60°C/10 s; 72°C/20 s for the Lightcycler and 50°C 2 min; 95°C/20 s; [95°C/1 s–60°C/25 s]×40 for the ABI instrument), followed by a fusion cycle in order to analyze the melting curve of the PCR products. Negative control without the reverse transcriptase was introduced to verify the absence of genomic DNA contaminants. Primers (see Table S2) were defined within exons (for mRNA-s) or in one intron and one exon (for pre-mRNA-s) using the PrimerSelect program of the Lasergene software (DNAStar). BLAST searches were performed to confirm gene specificity and the absence of multilocus matching at the primer site. The amplification efficiencies of primers were generated using the slopes of the standard curves obtained by a 10-fold dilution series of 4, with all experimental points falling within this range. The efficiency of the q-PCR amplifications for all pairs of primers is indicated in the table. Amplification specificity for each q-PCR reaction was confirmed by dissociation curve analysis. Determined Ct values (see Table S2) were then used for quantification, with the *tubulin* gene as reference. Each sample measurement was made at least in duplicate (technical replicate).

### Immunolabeling of Adult Brains

Experiments were done on whole-mounted adult brains as previously described [46]. Primary antibodies were rabbit anti-PER [66] at 1∶15,000, guinea pig GP47 anti-CLK [26] at 1∶15,000, mouse anti-PDF (Developmental Studies Hybridoma Bank) at 1∶50,000, and rabbit anti-CK2α. (Abcam, ab81435) at 1∶100. Secondary goat antibodies (Life Technologies) were Alexa 647- or Alexa 594-conjugated anti-rabbit at 1∶5,000, Alexa 488- or Alexa 647-conjugated anti-guinea pig at 1∶2,000, and Alexa 594- or Alexa 488-conjugated anti-mouse at 1∶2,000. Fluorescence signals were analyzed with a Zeiss AxioImager Z1 microscope with an ApoTome structured illumination module and an AxioCam MRm digital camera. Images for subcellular localization of CK2α were acquired with a Zeiss LSM-700 confocal microscope. Fluorescence intensity of individual cells was quantified from digital images of single focal planes with the NIH ImageJ software. We calculated a fluorescence index: I = 1 00(S-B)/B, which gives the fluorescence percentage above background (S (Signal) is fluorescence intensity and B (Background) is average intensity of the region adjacent to the positive cell). Index values were then averaged for the four PDF-positive s-LNv cells of 12–20 brain hemispheres for each time point.

## Supporting Information

Figure S1 **CLK degradation is accelerated in** ***tim > Tik*** **flies.** (A–E) Western blot of head extracts from flies collected at DD1. Time (h) is indicated as CT. Gray and black bars represent subjective day and subjective night, respectively. A CB stained band in the size range of CLK is used as a loading control for blots run on 4% gels. Brackets indicate hypo- and hyperphosphorylated forms of CLK. At least two independent experiments were performed for each blot. (A) Western blot of CLK protein in sonicated extracts of the indicated genotypes. Two representative examples are shown in addition to Figure 1A. (B) Western blot of CLK, PER, and TIM proteins as in (A) but from nonsonicated extracts of the indicated genotypes. (C) Sonicated extracts from the indicated genotypes were treated with or without λ protein phosphatase at the respective temperatures, and CLK protein was detected by Western blot. (D, Left) Western blot of CLK protein in nonsonicated head extracts from the indicated genotypes collected on the first day of constant darkness. CLK protein is shown on the immunoblot. A CB stained band in the size range of CLK is used as a loading control. (Right) CLK protein/*Clk* mRNA ratio of the indicated genotypes. Values from quantification of CLK bands of the left panel were divided with the values of RT-qPCR from Figure 1B. *w; tim-gal4* at CT2 was set to 100. (E) *CkIIα* RNAi decreases CK2α protein abundance. Samples were run on a 4–12% Bis-Tris gel. Anti-CK2α primary antibody was used for the blot. One copy of *tim-gal4* and two copies of the *CkIIα* RNAi construct were used for the experimental genotype.
(PDF)

Figure S2 **CK2 affects CLK in a** ***per^0^*** **background and impacts on** ***Clk*** **expression posttranscriptionally.** (A, B, D) Western blot of head extracts from flies collected at DD1. Time (h) is indicated as CT. A CB stained band in the size range of CLK is used as a loading control for blots run on 4% gels. Brackets indicate hypo- and hyperphosphorylated forms of CLK. At least two independent experiments were performed for each blot. (A) Comparison of CLK phosphorylation states between *per^+^tim^+^* sonicated extracts and their *per^0^ tim^0^* counterparts. We loaded 100 µg protein. *a*, *b*, and *c* are different protein extracts from the same genotype at the same time point. *w; tim-gal4/+*(*tim-gal4/+*), *per^0^ w*; *tim^0^* (*per^0^ tim^0^*), and *per^0^ w*; *tim^0^ tim-gal4; UAS-CkIIα^Tik^* (*per^0^ tim^0^ CkIIα^Tik^*) were used. (B, Left) Comparison between *tim>Tik* and controls in a *per^0^* background for TIM and CLK [*per^0^w; tim-gal4* (*per^0^ +*), *per^0^w; tim-gal4; UAS-CkIIα^Tik^* (*per^0^ CkIIα^Tik^*)]. *a* and *b* are different nonsonicated protein extracts from the same genotype at the same time point. We loaded 100 µg of extracts. Extracts were run on a 3–8% Tris-Acetate gel for TIM. (Middle) Quantitative RT-PCR measurements of *Clk* mRNA levels in heads of flies collected at DD1. Results are means of pooled values from two time points (CT2 and 14) with at least two independent samples for each time point. Error bars indicate s.e.m. Average values were normalized to the mean of the control (*per^0^ w*; *tim-gal4*) set to 100. Previous analysis of separate values at CT2 and CT14 indicated that they were similar (Table S1), justifying their common treatment (see above). (Right) CLK protein/*Clk* mRNA ratio of the indicated genotypes. Values from quantification of CLK bands of the left panel were divided with the values of RT-qPCR from the middle panel. *per^0^w; tim-gal4* was set to 100. (C) Quantitative RT-PCR measurements of *Clk* pre-mRNA levels in head extracts of flies collected at DD1. Average values from three independent experiments were normalized to the mean of the control (*w; tim-gal4*) at CT0 set to 100. Error bars represent s.e.m. (D) Comparison between *tim>Tik* and controls in a *per^0^* background for the VRI and PDP1ε proteins. Samples were resolved on a 3–8% Tris-Acetate gel. *per^0^w; tim-gal4* (*per^0^ +*) and *per^0^w; tim-gal4; UAS-CkIIα^Tik^* (*per^0^ CkIIα^Tik^*) were used. (E) Quantitative RT-PCR measurements of *cry* mRNA levels in heads of flies collected at DD1. Results are means of pooled values from two time points (CT2 and 14, which gave similar values) with at least two independent samples for each time point. Error bars indicate s.e.m. Average values were normalized to the control (*per^0^ w*; *tim-gal4*) average values set to 100.
(PDF)

Figure S3 ***CkIIβ*** **RNAi decreases CK2β protein abundance and causes PER and TIM accumulation.** (A) Western blot of CK2β. Extracts of *tim> CkIIβ RNAi* (*w; tim-gal4/106845; 32377/+*) and *tim-gal4/+* controls in a *per^+^* background were run on a 4–12% Bis-Tris gel. VIIa, d, and c indicate different isoforms of CK2β [64]. (B) Quantification of CLK, PER, and TIM signal intensity on Western blots in *tim> CkIIβ RNAi* and *tim-gal4/+* controls at six time points of DD1. Two independent experiments were quantified. Error bars stand for the difference of the respective values from each experiment and their mean. The intensities were normalized to the signal of a CB stained band. The highest intensity signal in *w*;*tim-gal4/+* was set to 100.
(PDF)

Figure S4 **CK2α overexpression induces a delay in TIM oscillation.** (A) Western blot of nonsonicated head extracts from flies collected at DD1. A CB stained band in the size range of CLK is used as a loading control. *tim > CkIIα* and *tim-gal4/+* controls are compared for TIM and PER. (B) Quantification of PER and TIM signal intensity from the Western blot in (A). The highest intensity signal in *w*;*tim-gal4/+* was set to 100. (C) CK2 phosphorylates CLK *in vitro*. (Top) Wild-type CLK was translated with an N-terminal 6-histidine fusion tag *in vitro*, affinity purified either in the absence or presence of PER and TIM, and subjected to phosphorylation assays by incubation with γ–^32^P-ATP either in the absence (−) or presence (+) of CK2. Intensity of incorporated ^32^P-phosphate into CLK (^32^P) was analyzed by autoradiography, and total CLK protein levels (CLK) were determined by Western blot analysis. (Bottom) Quantification of CLK-incorporated ^32^P-phosphate after normalization toward total CLK protein levels. Average CLK phosphorylation from at least three experiments ± s.e.m. are shown in the figure with wild-type CLK set to 100.
(PDF)

Figure S5 ***per*** **and** ***tim*** **transcription in** ***tim > Tik*** **and** ***tim > CkIIβ RNAi*** **animals.** (A) Quantitative RT-PCR measurements of *per* and *tim* mRNA levels in head extracts of flies collected at CT2. *tim>Tik* and controls are compared in a *per^0^ tim^0^* background. Mean mRNA levels +/− s.e.m. from at least three independent experiments are shown. Average values were normalized to the control mean (*per^0^ tim^0^*) set to 100. Genotypes: *per^0^ w*; *tim^0^* (*per^0^ tim^0^*) and *per^0^ w*; *tim^0^ tim-gal4; UAS-CkIIα^Tik^* (*per^0^ tim^0^ CkIIα^Tik^*). (B) Quantitative RT-PCR measurements of *per* and *tim* mRNA levels in *tim > CkIIß-RNAi* and control flies. Values were normalized to the maximum value (control at CT12) set to 100. Mean mRNA levels +/− s.e.m. from at least three independent experiments are shown.
(PDF)

Table S1 **Mean values of quantitative RT-PCR results with associated s.e.m.** Relative values of mRNA abundance measured by quantitative RT-PCR (see Material and Methods) are indicated for samples on *per^0^* or *tim^0^* background collected at CT2 and CT14. Mean levels are normalized to the highest value in the control genotype (*per^0^w;tim-gal4* or *w;tim^0^ tim-gal4;*) set to 100. The number of independent samples for each time point is shown in Figure 2 and Figure 6.
(DOCX)

Table S2 **qPCR primer specifications.** *This pair of primers was used in Figure 6B and Figure S2C. **These two pairs of primers were used in Figure 6B. Efficiency (E), DNA concentration ratio between cycles n+1 and n (1<E<2). R^2^, coefficient of determination of the calibration curve. Ct, Cycle threshold.
(DOCX)

## Abbreviations

CT: circadian time
DD: constant darkness
LD: light:dark
