# Supplementary figures and images for: The CK2 Kinase Stabilizes CLOCK and Represses Its Activity in the Drosophila Circadian Oscillator
Source: PLoS Biol. 2013 Aug 27;11(8):e1001645. doi: 10.1371/journal.pbio.1001645 (PMC3754892; doi:10.1371/journal.pbio.1001645)

**A**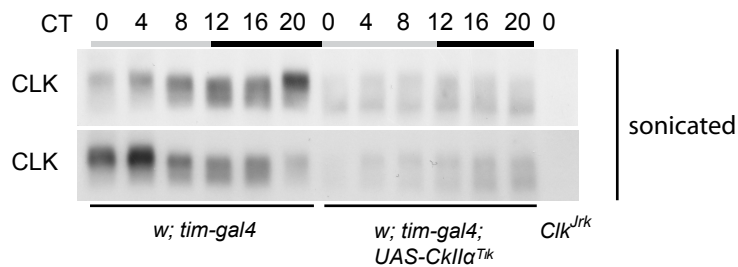**B**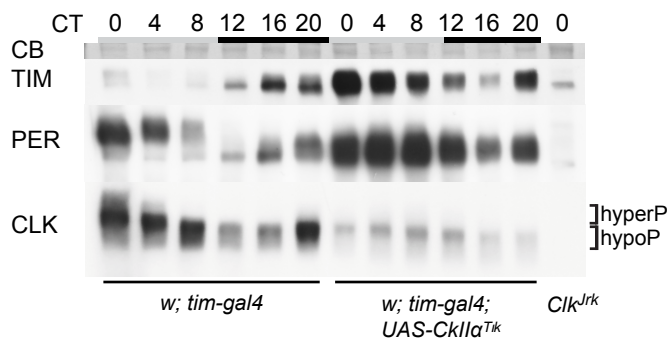**C**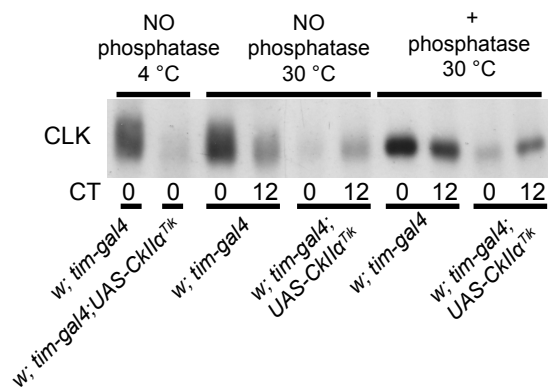**D**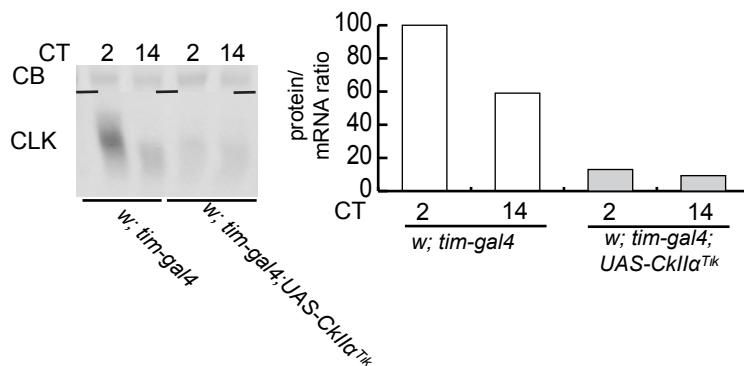**E**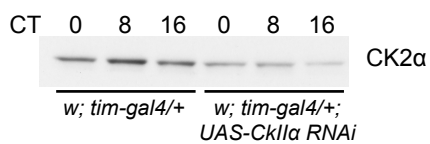

Supplement: Figure S1 — CLK degradation is accelerated in tim > Tik flies. (A–E) Western blot of head extracts from flies collected at DD1. Time (h) is indicated as CT. Gray and black bars represent subjective day and subjective night, respectively. A CB stained band in the size range of CLK is used as a loading control for blots run on 4% gels. Brackets indicate hypo- and hyperphosphorylated forms of CLK. At least two independent experiments were performed for each blot. (A) Western blot of CLK protein in sonicated extracts of the indicated genotypes. Two representative examples are shown in addition to Figure 1A. (B) Western blot of CLK, PER, and TIM proteins as in (A) but from nonsonicated extracts of the indicated genotypes. (C) Sonicated extracts from the indicated genotypes were treated with or without λ protein phosphatase at the respective temperatures, and CLK protein was detected by Western blot. (D, Left) Western blot of CLK protein in nonsonicated head extracts from the indicated genotypes collected on the first day of constant darkness. CLK protein is shown on the immunoblot. A CB stained band in the size range of CLK is used as a loading control. (Right) CLK protein/Clk mRNA ratio of the indicated genotypes. Values from quantification of CLK bands of the left panel were divided with the values of RT-qPCR from Figure 1B. w; tim-gal4 at CT2 was set to 100. (E) CkIIα RNAi decreases CK2α protein abundance. Samples were run on a 4–12% Bis-Tris gel. Anti-CK2α primary antibody was used for the blot. One copy of tim-gal4 and two copies of the CkIIα RNAi construct were used for the experimental genotype. (PDF) [file pbio.1001645.s001.pdf]

**A**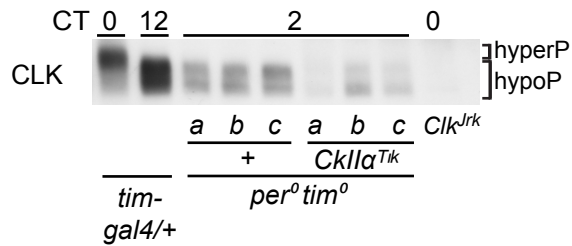**B**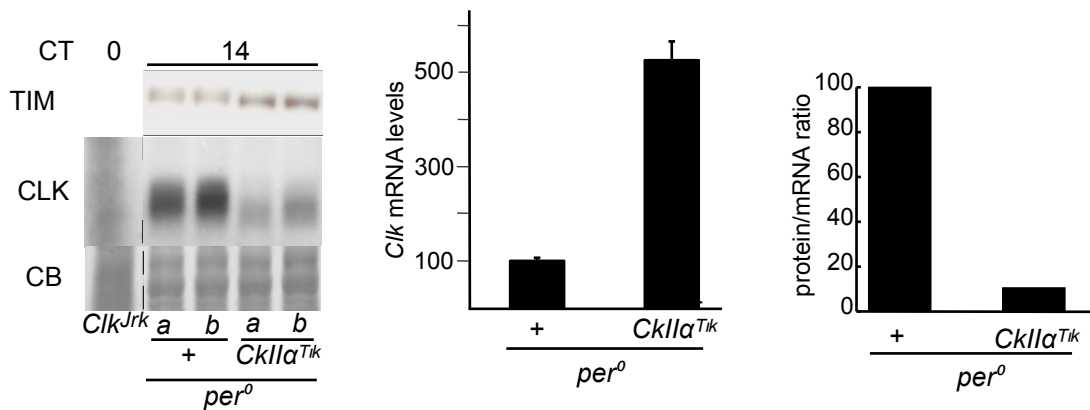**C**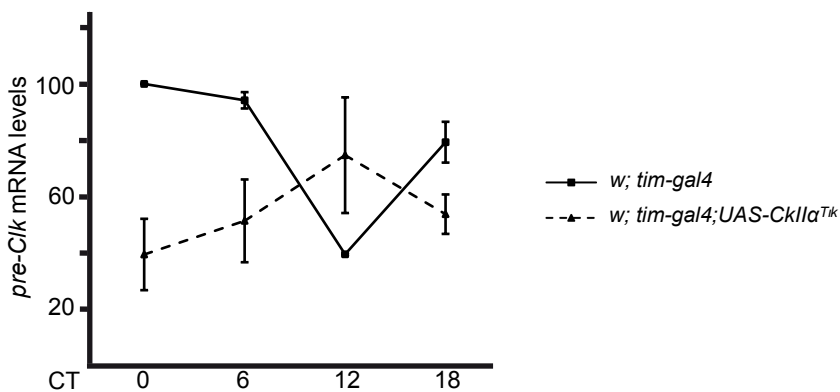**D**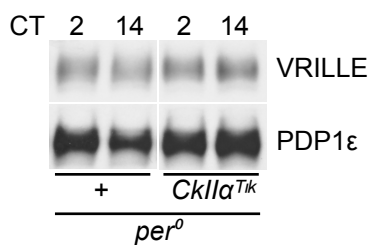**E**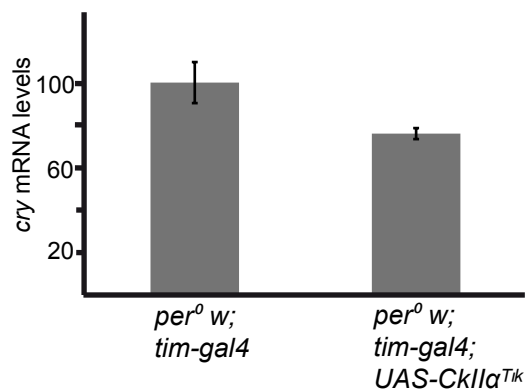

Supplement: Figure S2 — CK2 affects CLK in a per0 background and impacts on Clk expression posttranscriptionally. (A, B, D) Western blot of head extracts from flies collected at DD1. Time (h) is indicated as CT. A CB stained band in the size range of CLK is used as a loading control for blots run on 4% gels. Brackets indicate hypo- and hyperphosphorylated forms of CLK. At least two independent experiments were performed for each blot. (A) Comparison of CLK phosphorylation states between per+tim+ sonicated extracts and their per0 tim0 counterparts. We loaded 100 µg protein. a, b, and c are different protein extracts from the same genotype at the same time point. w; tim-gal4/+(tim-gal4/+), per0 w; tim0 (per0 tim0), and per0 w; tim0 tim-gal4; UAS-CkIIαTik (per0 tim0 CkIIαTik) were used. (B, Left) Comparison between tim>Tik and controls in a per0 background for TIM and CLK [per0w; tim-gal4 (per0 +), per0w; tim-gal4; UAS-CkIIαTik (per0 CkIIαTik)]. a and b are different nonsonicated protein extracts from the same genotype at the same time point. We loaded 100 µg of extracts. Extracts were run on a 3–8% Tris-Acetate gel for TIM. (Middle) Quantitative RT-PCR measurements of Clk mRNA levels in heads of flies collected at DD1. Results are means of pooled values from two time points (CT2 and 14) with at least two independent samples for each time point. Error bars indicate s.e.m. Average values were normalized to the mean of the control (per0 w; tim-gal4) set to 100. Previous analysis of separate values at CT2 and CT14 indicated that they were similar (Table S1), justifying their common treatment (see above). (Right) CLK protein/Clk mRNA ratio of the indicated genotypes. Values from quantification of CLK bands of the left panel were divided with the values of RT-qPCR from the middle panel. per0w; tim-gal4 was set to 100. (C) Quantitative RT-PCR measurements of Clk pre-mRNA levels in head extracts of flies collected at DD1. Average values from three independent experiments were normalized to the mean [file pbio.1001645.s002.pdf]

**A**

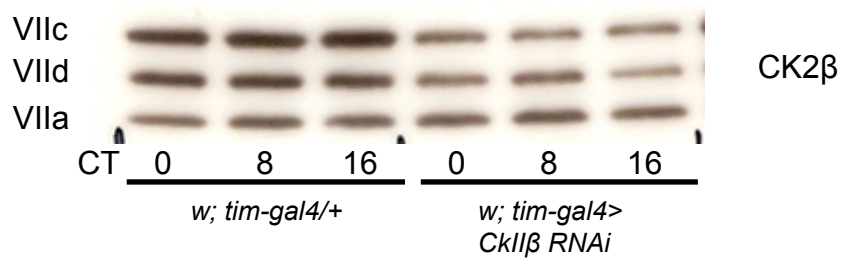

**B**

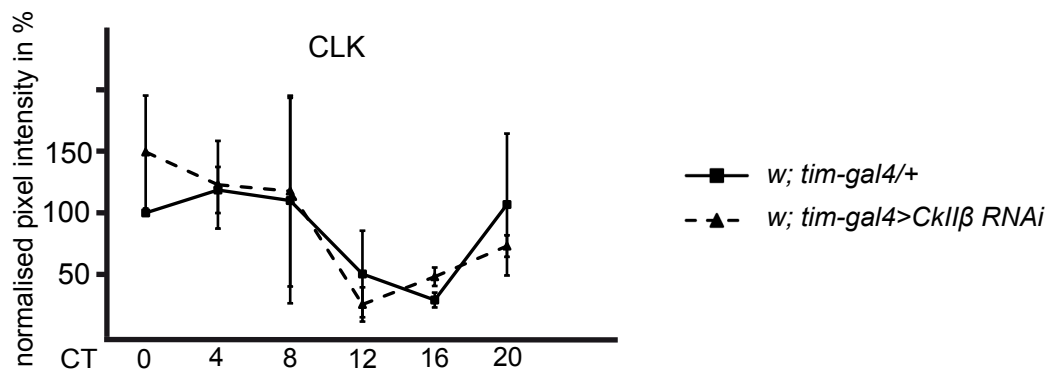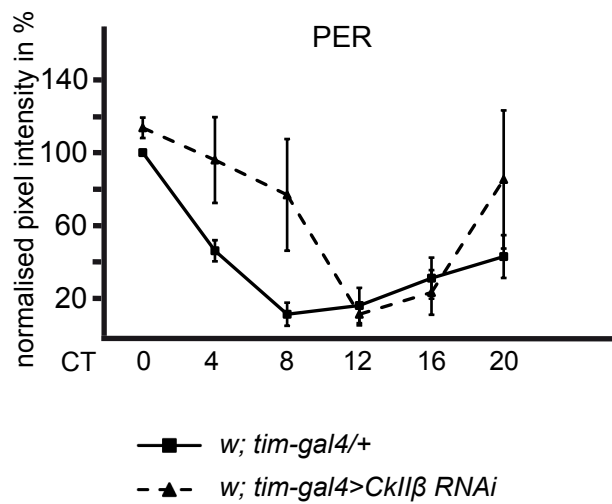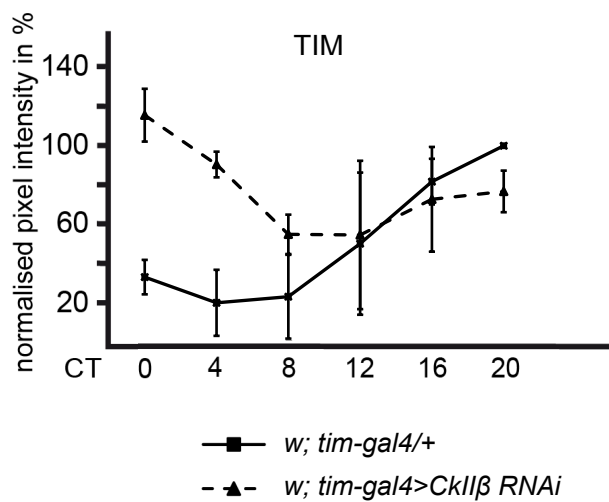

Supplement: Figure S3 — CkIIβ RNAi decreases CK2β protein abundance and causes PER and TIM accumulation. (A) Western blot of CK2β. Extracts of tim> CkIIβ RNAi (w; tim-gal4/106845; 32377/+) and tim-gal4/+ controls in a per+ background were run on a 4–12% Bis-Tris gel. VIIa, d, and c indicate different isoforms of CK2β [64]. (B) Quantification of CLK, PER, and TIM signal intensity on Western blots in tim> CkIIβ RNAi and tim-gal4/+ controls at six time points of DD1. Two independent experiments were quantified. Error bars stand for the difference of the respective values from each experiment and their mean. The intensities were normalized to the signal of a CB stained band. The highest intensity signal in w;tim-gal4/+ was set to 100. (PDF) [file pbio.1001645.s003.pdf]

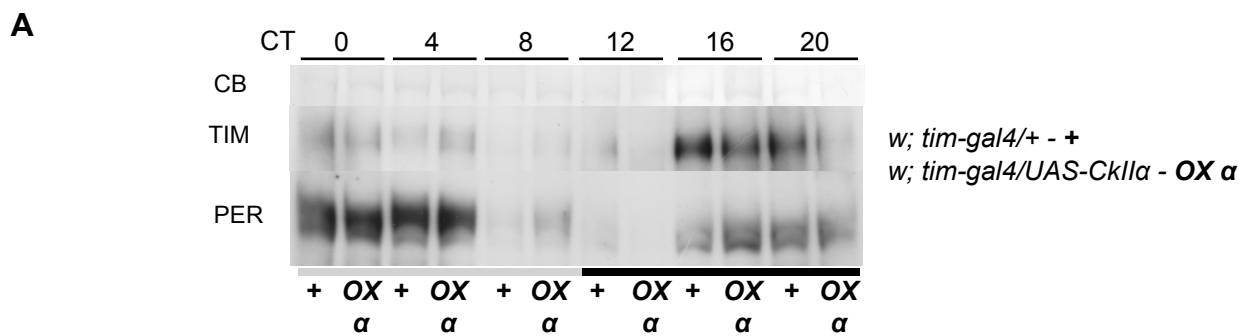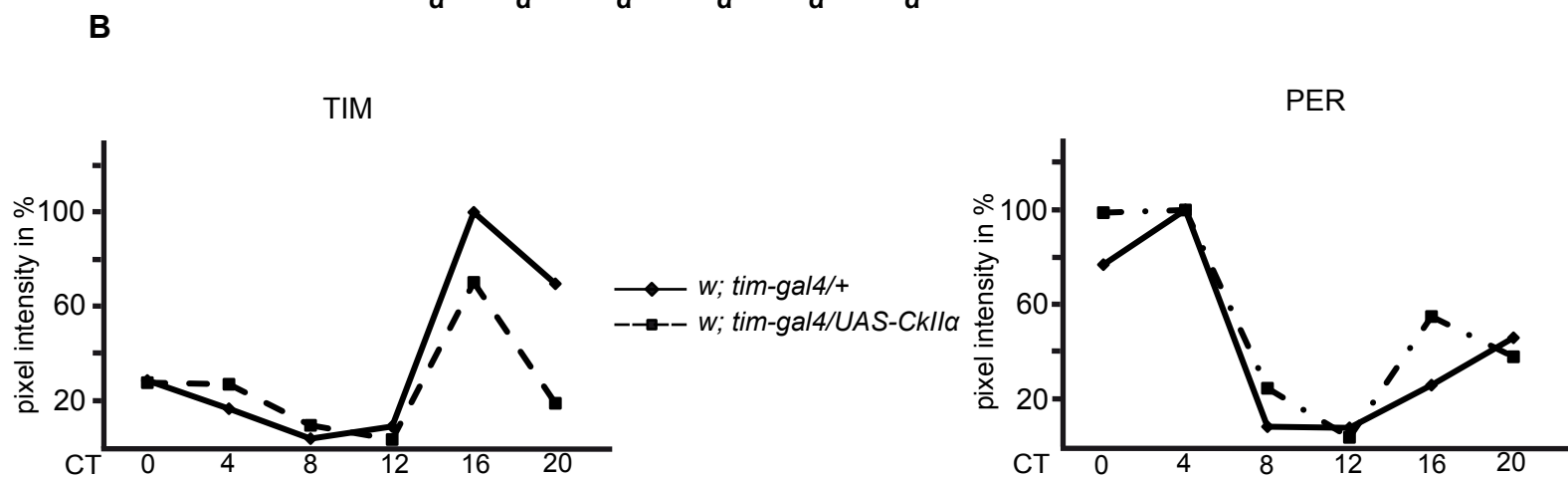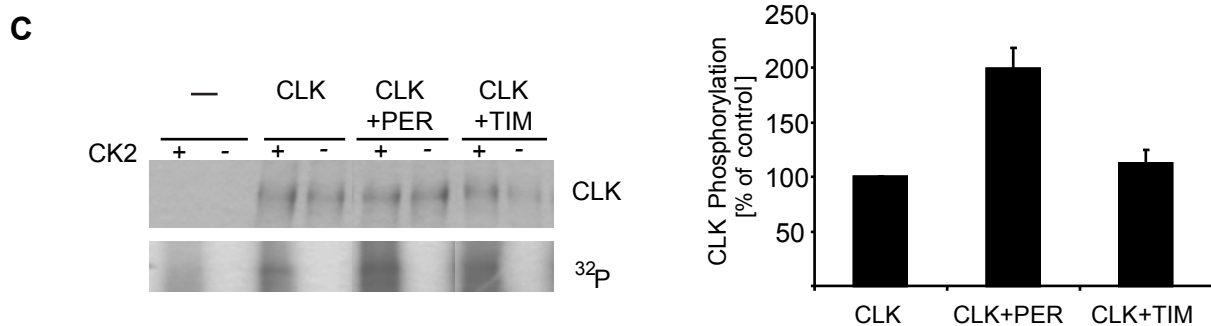

Supplement: Figure S4 — CK2α overexpression induces a delay in TIM oscillation. (A) Western blot of nonsonicated head extracts from flies collected at DD1. A CB stained band in the size range of CLK is used as a loading control. tim > CkIIα and tim-gal4/+ controls are compared for TIM and PER. (B) Quantification of PER and TIM signal intensity from the Western blot in (A). The highest intensity signal in w;tim-gal4/+ was set to 100. (C) CK2 phosphorylates CLK in vitro. (Top) Wild-type CLK was translated with an N-terminal 6-histidine fusion tag in vitro, affinity purified either in the absence or presence of PER and TIM, and subjected to phosphorylation assays by incubation with γ–32P-ATP either in the absence (−) or presence (+) of CK2. Intensity of incorporated 32P-phosphate into CLK (32P) was analyzed by autoradiography, and total CLK protein levels (CLK) were determined by Western blot analysis. (Bottom) Quantification of CLK-incorporated 32P-phosphate after normalization toward total CLK protein levels. Average CLK phosphorylation from at least three experiments ± s.e.m. are shown in the figure with wild-type CLK set to 100. (PDF) [file pbio.1001645.s004.pdf]

**A**

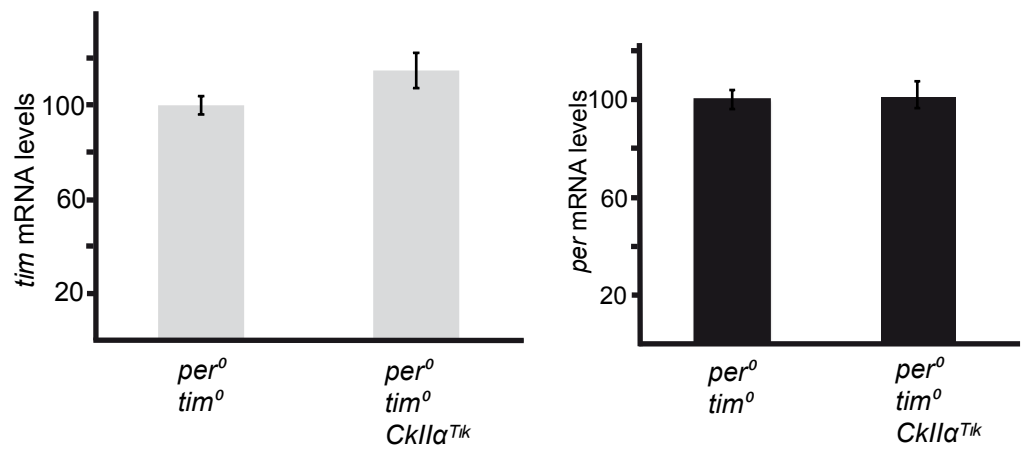

**B**

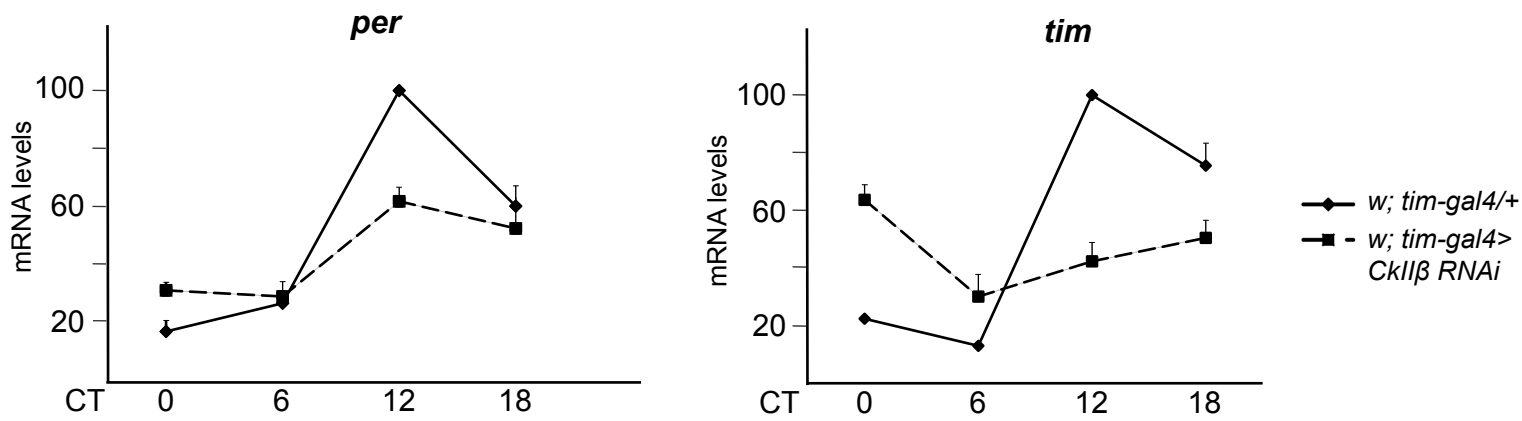

Supplement: Figure S5 — per and tim transcription in tim > Tik and tim > CkIIβ RNAi animals. (A) Quantitative RT-PCR measurements of per and tim mRNA levels in head extracts of flies collected at CT2. tim>Tik and controls are compared in a per0 tim0 background. Mean mRNA levels +/− s.e.m. from at least three independent experiments are shown. Average values were normalized to the control mean (per0 tim0) set to 100. Genotypes: per0 w; tim0 (per0 tim0) and per0 w; tim0 tim-gal4; UAS-CkIIαTik (per0 tim0 CkIIαTik). (B) Quantitative RT-PCR measurements of per and tim mRNA levels in tim > CkIIß-RNAi and control flies. Values were normalized to the maximum value (control at CT12) set to 100. Mean mRNA levels +/− s.e.m. from at least three independent experiments are shown. (PDF) [file pbio.1001645.s005.pdf]
