# Supplementary material for: The CK2 Kinase Stabilizes CLOCK and Represses Its Activity in the Drosophila Circadian Oscillator
Source: PLoS Biol. 2013 Aug 27;11(8):e1001645. doi: 10.1371/journal.pbio.1001645 (PMC3754892; doi:10.1371/journal.pbio.1001645)
Supplement: Table S1 — Mean values of quantitative RT-PCR results with associated s.e.m. Relative values of mRNA abundance measured by quantitative RT-PCR (see Material and Methods) are indicated for samples on per0 or tim0 background collected at CT2 and CT14. Mean levels are normalized to the highest value in the control genotype (per0w;tim-gal4 or w;tim0 tim-gal4;) set to 100. The number of independent samples for each time point is shown in Figure 2 and Figure 6. (DOCX) [file pbio.1001645.s006.docx]

|  | *per^0^w;tim-gal4* | | *per^0^w;tim-gal4;UAS-CkIIα^Tik^* | | *w;tim^0^ tim-gal4;* | | *w;tim^0^ tim-gal4; UAS-CkIIα^Tik^* | |
| --- | --- | --- | --- | --- | --- | --- | --- | --- |
|  | CT2 | CT14 | CT2 | CT14 | CT2 | CT14 | CT2 | CT14 |
| *Clk* mRNA |  |  |  |  |  |  |  |  |
| mean | 100.0 | 79.4 | 516.9 | 427.6 | 72.1 | 100.0 | 293.1 | 463.1 |
| s.e.m. | 7.3 | 6.2 | 26.0 | 55.2 | 8.2 | 13.9 | 37.0 | 97.0 |
|  |  |  |  |  |  |  |  |  |
|  |  |  |  |  |  | |  | |
| *tim* mRNA |  |  |  |  |  |  |  |  |
| mean | 100.0 | 97.9 | 89.4 | 94.5 | 100.0 | 98.2 | 125.3 | 103.3 |
| s.e.m. | 7.5 | 8.1 | 16.4 | 8.2 | 8.4 | 9.7 | 5.8 | 11.7 |
|  |  |  |  |  |  |  |  |  |
|  |  | |  | |  | |  | |
| *per* mRNA |  |  |  |  |  |  |  |  |
| mean | 79.6 | 100.0 | 69.3 | 72.9 | 100.0 | 98.4 | 90.1 | 104.1 |
| s.e.m. | 14.9 | 24.3 | 12.5 | 8.1 | 15.8 | 11.4 | 12.1 | 27.6 |
