# Supplementary material for: The CK2 Kinase Stabilizes CLOCK and Represses Its Activity in the Drosophila Circadian Oscillator
Source: PLoS Biol. 2013 Aug 27;11(8):e1001645. doi: 10.1371/journal.pbio.1001645 (PMC3754892; doi:10.1371/journal.pbio.1001645)
Supplement: Table S2 — qPCR primer specifications. *This pair of primers was used in Figure 6B and Figure S2C. **These two pairs of primers were used in Figure 6B. Efficiency (E), DNA concentration ratio between cycles n+1 and n (1<E<2). R2, coefficient of determination of the calibration curve. Ct, Cycle threshold. (DOCX) [file pbio.1001645.s007.docx]

| Transcript (exons) | 5' primer | 3' primer | Amplicon size | Efficiency | R^2^ | Ct |
| --- | --- | --- | --- | --- | --- | --- |
|  |  |  |  |  |  |  |
| *tubulin** | TCCAATAAAAACTCAATATGCGTGA | CAAGCAGTAGAGCTCCCAGCA | 94 bp | 1.94 | 0.99 | 20-21 |
| *tubulin* (E1-E2) | TCCTTGTCGCGTGTGAAACA | CCGAACGAGTGGAAGATGAG | 464 bp | 1.93 | 0.99 | 20-21 |
| *period *** | GAGCAGCTACAAGGTTCCCG | CCACGTGCGATATGATCCC | 91 bp | 1.98 | 0.99 | 23-26 |
| *period* (E6-E7) | ACCCGCATCCTTCGCTTTTCTACA | AGGTGAGCCAGTGGTGAGGACGGG | 219 bp | 1.91 | 0.99 | 25-27.5 |
| *pre-period* | AGCTGGGCGGCACAATACTA | CGCTGTTGCATCAACGTGAG | 197 bp | 1.94 | 0.99 | 27.5-29.5 |
| *timeless*** | TCAAGAATTTGGGAAGCGGA | GCGACCAAGAGCAAACGGTA | 174 bp | 1.98 | 0.99 | 20-23 |
| *timeless* (E12-E13) | AGTTGGTCATGCGCAGCAAATG | TCCTTTTCGTACACAGATGCCA | 446 bp | 1.87 | 0.99 | 19.5-25 |
| *pre-timeless* | ACTGCTTTCCAATGCGGTATG | AAGCCTCCGAAAAACATATGAAAA | 172 bp | 1.96 | 0.99 | 23.5-28 |
| *Clock* (E7-E8) | CCAAATAAATCCACTGAAAACAT | GAGAGGCACCATTTTCGGAGTAC | 369 bp | 1.85 | 0.99 | 23-28 |
| *pre-Clock* | CGTAGGTAGCCGTGCAGAGAGT | CGCAGGTTGTCCATTTGAGC | 121 bp | 2.00 | 0.99 | 26.5-28 |
| *cryptochrome* (E3-E4) | GTACGTCCCGGAGTTGATGAATGT | CACGTCGGCCAGCCAGAAGAACT | 253 bp | 1.90 | 0.99 | 20.5-27 |
|  |  |  |  |  |  |  |
